# CD32^+^CD4^+^ T Cells Sharing B Cell Properties Increase With Simian Immunodeficiency Virus Replication in Lymphoid Tissues

**DOI:** 10.3389/fimmu.2021.695148

**Published:** 2021-06-16

**Authors:** Nicolas Huot, Philippe Rascle, Cyril Planchais, Vanessa Contreras, Caroline Passaes, Roger Le Grand, Anne-Sophie Beignon, Etienne Kornobis, Rachel Legendre, Hugo Varet, Asier Saez-Cirion, Hugo Mouquet, Beatrice Jacquelin, Michaela Müller-Trutwin

**Affiliations:** ^1^ Institut Pasteur, Unité HIV, Inflammation et Persistance, Paris, France; ^2^ Université Paris Diderot, Sorbonne Paris Cité, Paris, France; ^3^ Institut Pasteur, INSERM U1222, Laboratoire d’Immunologie Humorale, Paris, France; ^4^ CEA-Université Paris Sud-Inserm, U1184, IDMIT Department, IBFJ, Fontenay-aux-Roses, France; ^5^ Hub de Bioinformatique et Biostatistique - Département Biologie Computationnelle, Institut Pasteur, Paris, France; ^6^ Plate-forme Technologique Biomics – Centre de Ressources et Recherches Technologiques (C2RT), Institut Pasteur, Paris, France

**Keywords:** SIV, HIV, CD4, CD20, LN, intestine, natural host, CD32

## Abstract

CD4 T cell responses constitute an important component of adaptive immunity and are critical regulators of anti-microbial protection. CD4^+^ T cells expressing CD32a have been identified as a target for HIV. CD32a is an Fcγ receptor known to be expressed on myeloid cells, granulocytes, B cells and NK cells. Little is known about the biology of CD32^+^CD4^+^ T cells. Our goal was to understand the dynamics of CD32^+^CD4^+^ T cells in tissues. We analyzed these cells in the blood, lymph nodes, spleen, ileum, jejunum and liver of two nonhuman primate models frequently used in biomedical research: African green monkeys (AGM) and macaques. We studied them in healthy animals and during viral (SIV) infection. We performed phenotypic and transcriptomic analysis at different stages of infection. In addition, we compared CD32+CD4+ T cells in tissues with well-controlled (spleen) and not efficiently controlled (jejunum) SIV replication in AGM. The CD32^+^CD4^+^ T cells more frequently expressed markers associated with T cell activation and HIV infection (CCR5, PD-1, CXCR5, CXCR3) and had higher levels of actively transcribed SIV RNA than CD32^-^CD4^+^T cells. Furthermore, CD32^+^CD4^+^ T cells from lymphoid tissues strongly expressed B-cell-related transcriptomic signatures, and displayed B cell markers at the cell surface, including immunoglobulins CD32+CD4+ T cells were rare in healthy animals and blood but increased strongly in tissues with ongoing viral replication. CD32^+^CD4^+^ T cell levels in tissues correlated with viremia. Our results suggest that the tissue environment induced by SIV replication drives the accumulation of these unusual cells with enhanced susceptibility to viral infection.

## Introduction

HIV-infected individuals mount immune responses resulting in a decrease of viral load by the end of the acute infection but, even in HIV controllers, the host is not able of clearing the infection. Combined antiretroviral therapy (ART) has changed HIV infection from a lethal disease into a manageable chronic infection. Indeed, ART efficiently controls HIV replication leading to undetectable virus in the blood and considerably increasing the life expectancy of people living with HIV (PLH). However, the virus persists in cellular and anatomical reservoirs, from which the virus most often rapidly rebounds in case of ART interruption (1–3). Although tremendous progress has been made in our understanding of HIV biology and pathogenesis, the composition and dynamics of the viral reservoir and the mechanisms of HIV persistence remain ill-defined.

Studies in non-human primates (NHP) infected with SIVmac have shown that viral seeding occurs in the first hours and days post-infection (4, 5). Several factors can influence the establishment and persistence of HIV/SIV reservoirs, such as the timing of initiation of ART. When ART is initiated in primary infection, the subsequent long-term decline of the reservoir is stronger than if ART is initiated in chronic infection (6, 7). Immune activation could also modify viral seeding as well as related cell trafficking (8). Moreover, HIV reservoir cells can hide from the immune system by residing in anatomical sanctuaries (1, 9–11). Follicular helper T cells (T_FH_) in B cell follicles of secondary lymphoid tissues (SLT) as well as Treg cells (CTLA-4^+^ CD4^+^ T cells) in the T zone of SLT have been shown to be potential major reservoirs of HIV/SIV viruses during ART (12–16). Many efforts have been made to identify cellular markers specific to reservoir cells, in particular latently infected cells, i.e. cells harboring viral DNA in the cellular genome without expressing viral proteins. It is though not excluded that low-level viral replication contributes to the viral reservoir. It has been shown that CD4^+^ T cells expressing high CD2 surface levels harbor higher HIV DNA copy numbers (range, 3- to 10.8-fold) compared to total CD4^+^ T cells (17). It has also been shown that cells expressing exhaustion markers such as PD-1, TIGIT, and LAG-3 were positively associated with the frequency of CD4^+^ T cells harboring HIV DNA (18). Memory CD4^+^ T cells co-expressing these three markers were up to 10-fold enriched for HIV compared to total CD4^+^ T cells (18). In the blood of HIV-infected individuals on suppressive ART for more than 3 years, CXCR3^+^CCR6^+^ central memory CD4^+^ T cells were shown to contain the highest amount of integrated HIV DNA and the lowest ratio of cell-associated (ca)-unspliced HIV RNA to DNA compared to all T-cell subsets studied (19). Blood CXCR3-expressing CD4 T cells represented the major blood compartment containing inducible replication-competent virus in treated aviremic HIV-infected individuals (20). CD30^+^CD4^+^ T cells have been shown to be enriched for ca-HIV RNA (21). However, this enrichment was not observed in all studied individuals, and CD30^+^CD4^+^ T cells were not significantly enriched for HIV DNA. CD20, normally expressed on B cells, has recently been described as a marker for HIV-infected cells in patients (22, 23). The use of latent reversal agents and anti-CD20 monoclonal antibody therapy allowed the depletion of a part of HIV-reservoir cells after viral reactivation *ex vivo* (23). CD32a (FcγRIIa)—a low-affinity IgG receptor known to be expressed on myeloid cells, granulocytes, NK cells and B cells (24), has been proposed as a surface marker for *in vitro* HIV-infected quiescent CD4^+^ T cells and for persistent HIV-infected CD4^+^ T cells in the blood of ART-treated PLH (25). Analyses of CD32^+^ CD4^+^ T cells in tissues from ART-treated PLH were associated with a T_FH_ phenotype, consistent with a role for CD32^+^CD4^+^ T cells in reservoir composition (26). Optimization of the CD32^+^CD4^+^ T cell purification protocol revealed significant enrichment for HIV DNA in these cells (27). Of note, several reports clearly showed that CD32 is not a marker for latently-infected CD4^+^ T cells *in vivo* (22, 28–30). It was rather demonstrated that CD32 expression on CD4^+^ T cells is frequently associated with actively transcribed virus (31). While CD32 is unlikely to be a marker of latently infected cells, the description of CD32^+^CD4^+^ T cells raises the question of the origin and the role CD32a^+^CD4^+^ T cells.

We addressed the question if a HIV infection favors the emergence of these cells. To determine the dynamics of CD32^+^CD4^+^ T cells in distinct tissues *in vivo*, we studied two nonhuman primate models frequently used in biomedical research: African green monkeys (AGM) and macaques. Asian species of NHP, such as rhesus and cynomolgus macaques (MAC), experimentally infected by SIVmac, experience a spectrum of disorders similar to those seen in HIV-1 infected humans (32). Nonhuman primates from Africa, such as AGM, sooty mangabeys, and mandrills, are natural hosts of SIV (33–35). SIV infection in these natural hosts generally does not lead to any signs of disease, even though they carry high plasma and intestinal viral load (36, 37). An important common feature of SIV infection in natural hosts is that these animals rapidly resolve inflammation and consistently exhibit lower levels of immune activation than PLH or MAC infected with SIV (36). Natural hosts of SIV also harbor extremely low levels of infected CD4^+^ T cells in lymph nodes (LN) and spleen in contrast to PLH and MAC infected with SIVmac (Huot et al., 2016). In this context, the difference in viral reservoir in SLT between the natural host and MAC could be useful to understand the dynamics of CD32^+^CD4^+^ T cells during HIV/SIV infection. We analyzed CD32^+^CD4^+^ T cells in healthy animals and during SIV infection. We performed phenotypic and transcriptomic characterization of CD32^+^CD4^+^ T cells at different stages of infection in lymphoid and non-lymphoid tissues (LN, spleen, jejunum, ileum, liver, blood). Since several studies suggested that these cells could correspond to doublets (28–30), we performed several controls including imaging experiments to ensure that the analyses were made on single cells. We show here that CD32^+^CD4^+^ T cells were not or only slightly increased in blood and liver, while strongly increased in LNs, spleen, and intestine during SIVmac infection. CD32^+^CD4^+^ T cells displayed higher levels of actively transcribed SIV RNA than CD32^-^CD4^+^ T cells in SLT and gut during both SIVmac and SIVagm chronic infection. CD32^+^CD4^+^ T cell levels correlated with viremia. CD32^+^CD4^+^ T cells from lymphoid tissues shared properties with B cells (CD20, IgG, IgM) and also expressed markers that were described to be often expressed on HIV infected and/or reservoir cells (CCR5, PD-1, CXCR5, CXCR3). Our results support the hypothesis that CD32^+^CD4^+^ T cells are a target for productive viral replication and suggest that general immune activation and local inflammation drives the accumulation of these peculiar cells with enhanced susceptibility to HIV infection.

## Material And Methods

### Monkeys and SIV Infections

African green monkeys (Caribbean *Chlorocebus sabaeus*, AGM) and cynomolgus macaques (*Macaca fascicularis*, MAC) were included in this study (**Supplementary Figure 1**). The AGM were infected with SIVagm.sab92018, and the MAC with SIVmac251 as previously described (38, 39). The viremia levels are shown in **Supplementary Table 1**. All SIV-infected MAC where viremic.

The AGM and MAC were housed at the IDMIT Center (Fontenay-aux-Roses, France). All experimental procedures were conducted in strict compliance with the international European guidelines 2010/63/UE for the protection of animals used for experimentation and other scientific purposes and the French law (French decree 2013-118). The IDMIT center complies with the Standards for Human Care and Use of the Office for Laboratory Animal Welfare (OLAW, USA) under OLAW Assurance number A5826-86. Monkeys were monitored under the supervision of the veterinarians in charge of the animal facilities. Animal experimental protocols were approved by the Ethical Committee of Animal Experimentation (CETEA-DSV, IDF, France) (Notification 12-098 and A17-044). The pVISCONTI study was approved and accredited under statement number A15-035 by the ethics committee “*Comité d’Ethique en Expérimentation Animale du CEA”*, registered and authorized under Number 2453-2015102713323361v2 by the French Ministry of Education and Research.

Monkeys were sedated before handling with Ketamine Chlorhydrate (Rhone-Mérieux, Lyons, France. The sample size varied from 3 to 9 monkeys per group (*n* = 6 in most experiments). Samples were collected in random order according to the tripartite harmonized International Council for Harmonization of Technical Requirements for Pharmaceuticals for Human Use (ICH) Guideline on Methodology (previously coded Q2B). The investigators were not blinded while the animal handlers were blinded to group allocation.

### Tissue Collections and Processing

Whole venous blood was collected in ethylenediaminetetraacetic acid (EDTA) tubes. Peripheral blood mononuclear cells (PBMCs) were isolated by Ficoll density-gradient centrifugation. Biopsies of peripheral LNs (pLN) were performed by excision. Other tissues were collected at autopsy. After careful removal of adhering connective and fat tissues, pLN, spleen, liver, and gut tissue were dissociated using the gentlemacS™ Dissociator technology (Miltenyi Biotec, Germany). Red blood cells were lysed in hypotonic solution and washed twice in PBS. The cell suspension was subsequently filtered through 100- and 40-μm cell strainers, and cells were washed with cold phosphate-buffered saline (PBS). Cells were either immediately stained for flow cytometry or cryopreserved in 90% foetal bovine serum (FBS) and 10% dimethyl sulfoxide (DMSO) and stored in liquid nitrogen vapour.

### Quantification of Viral Load

Viral RNA copy numbers in plasma and cell-associated (ca) viral DNA and RNA from the animals were quantified as previously described (38–40). For plasma, the cut-off value corresponded to 60 viral copies or below per ml of plasma. Ultrasensitive determinations of plasma viral loads were achieved by concentrating the virus from a larger volume of material available by ultracentrifugation. For the quantification of ca-viral DNA and RNA, total nucleic acids were extracted from cells sorted as described below. The number of cells analyzed for viral load was the same for each cellular fraction of each sample. The relative fold-change of SIV transcripts was determined using the delta-delta CT method normalized to the 18s RNA levels, as described (38).

### Production of Recombinant Anti-CD32 Monoclonal Antibody

IgH and IgL DNA fragments coding for human MDE8 (41) antibody were prepared by PCR-amplification from codon-optimized synthetic genes (Life Technologies, Thermo Fisher Scientific). Purified digested DNA fragments were cloned into human Igγ1-and Igλ-expressing vectors (42), and human MDE8 IgG1 antibod-ies were produced by transient co-transfection of Freestyle™ 293-F suspension cells (Thermo Fisher Scientific) using PEI-precipitation method as previously described (43). Recombinant IgG1 antibodies were purified by batch/gravity-flow affinity chromatog-raphy using protein G sepharose 4 fast flow beads (GE Healthcare, Chicago, IL) according to the manufacturer’s instructions, extensively dialyzed against PBS using Slide-A-Lyzer^®^ dial-ysis cassettes (Thermo Fisher Scientific) and quantified using NanoDrop 2000 instrument (Thermo Fisher Scientific) (43).

### Polychromatic Flow Cytometry

Cryopreserved cells were thawed in foetal bovine serum. Cryopreserved or freshly isolated cells were counted, examined for viability, and then incubated with Fcγ receptor blocker from Biolegend for 10 minutes. Undiluted extracellular antibody cocktail mix was added and incubated for 20 minutes. All incubations were performed in the dark at room temperature. If no intracellular staining was done, the cells were washed, fixed with a 4% paraformaldehyde solution and stored in the dark at 4°C until acquisition. Intracellular staining was performed as follows: after staining for cell surface molecules, cells were fixed/permeabilized using the “BD Cytofix/Cytoperm Plus Fixation/Permeabilization” kit. Intracellular markers were stained with the antibody cocktail for one hour. Finally, cells were fixed with a 4% paraformaldehyde solution and stored in the dark at 4°C until acquisition. All phenotyping data were acquired on a BD FACS LSR II (BD Biosciences) or on a LSR Fortessa (BD Immunocytometry Systems). The data were further analyzed using FlowJo 10.4.2 software (FlowJo, LLC, Ashland, OR, USA). *T*-SNE was performed using FlowJo isuali FlowJo, LLC, Ashland, OR, USA). The antibodies used are listed below: anti-CD45 (clone D058-1283, BD bioscience); anti-CD3 (clone SP34-2, BD bioscience); anti-CD4 (clone L200, BD bioscience); anti-CD32 (cloneFLI8.26)(BD bioscience); anti-CD20 (clone 2H7, (BD bioscience); anti-CD14 (clone TÜK4,M iltenyi Biotec); anti-CD28 (clone CD28.2, BD bioscience); anti-CD95 (clone DX2, BD bioscience), anti-CXCR3 (clone 1C6/CXCR3, BD bioscience); anti-CXCR5 (clone MU5UBEE, Ebioscience); anti-PD-1 (clone EH12.1, BD bioscience), anti-CXCR4 (clone 12G5,B D bioscience); anti-CCR5 (clone 3A9, BD bioscience); anti-CD86 (clone FUN-1, BD bioscience); anti-CD83 (clone HB15e, BD bioscience); anti-CD39 (clone A1, Biolegend); anti-CD25 (cloneM-A251, BD bioscience); anti-MHC-E (clone 3D12HLA-E, ebioscience), anti-TIM-3 (clone 7D3, BD bioscience); anti-CD8alpha (clone SK1, BD bioscience); anti-NKG2a/c (clone Z199, Beckman-Coulter); anti-IgG (cloneG18-145, BD bioscience); anti-IgM (clone G20-127, BD bioscience). The anti-CD32 antibody used recognizes CD32A and not the CD32B isoform.

### Immunofluorescence Staining

Purified splenic CD4 T cells were isolated from five MAC and five AGM, using a MACS negative selection CD4 T Cell Isolation Kit (Miltenyi Biotec, Boston, MA). Cells were then adhered to the poly-l-lysine-coated glass slides at 37°C in RPMI 1640 containing 10% FCS. After 2 hours, CD4 T cells were prepared for fixed cell immunofluorescent confocal microscopy. Briefly, cells were blocked with 5% heat-inactivated goat serum in PBS for 20 min at room temperature. Anti CD20-APC (clone 2H7), anti CD4-alexa700 clone (clone L200), and anti CD32 antibodies described in the method section were added to the culture for 1h. Antibodies were used in the range of 0.5–2 μg/ml. The slides were rinsed in PBS containing 2% FCS and the cells were fixed and permeabilized. A secondary goat α-human IgG conjugated to Alexa Fluor 488 (Molecular Probes, Grand Island, NY) was used to detect the anti-CD32 antibody. Slides were then covered with 0.15-mm coverslips (VWR Scientific, Philadelphia, PA), using mounting media (Vectashield, Burlingame, CA) containing DAPI (Invitrogen, Carlsbad, CA) to visualize nuclear chromatin. Two-dimensional micrographs were taken using a multilaser-based spinning disk confocal microscope (Zeiss).

### Cell Sorting of CD32^+^CD4^+^ T Cells

We performed cell sorting on frozen spleen and jejunum from chronically infected MAC or AGM. All incubations were performed in the dark at room temperature. Cells were thawed in 20% FBS-containing media supplemented with benzonase nuclease and counts and viabilities were performed (Life Technologies). Cells were incubated with Fcγ receptor blocker for 10 minutes. The antibody cocktail mix was added and incubated for 20 minutes. A viability dye for flow cytometry (LIVE/DEAD Fixable Dead Cell Stain Kit, Invitrogen) was included in all experiments to determine cell viability. The antibodies used are listed below: anti-CD45 (clone D058-1283) (BD bioscience); anti-CD3 (clone SP34-2) (BD bioscience); anti-CD4 (clone L200) (BD bioscience); anti-CD32 (cloneFLI8.26) (BD bioscience); anti-CD20 (clone 2H7, BD bioscience); anti-CD14 (clone TÜK4, Miltenyi Biotec); anti-CD8alpha (clone SK1, BD bioscience); anti-NKG2a/c (clone Z199, Beckman-Coulter). After surface staining, cells were washed and resuspended in complete medium and stored in the dark at 4°C until acquisition. Cells were sorted on a FACSAria II (BD Biosciences) in purity mode. The gating strategy is shown in **Supplemental Figure 1**. Doublet cells were excluded from the sorting using the FSC-H and FSC-A parameters. For RNA extraction, cells were directly collected in a lysis buffer containing TCEP (Qiagen). The purity of the cells was >97%.

### Genome-Wide RNA Sequencing

RNA was isolated from the sorted cell populations using the Rneasy^®^ Mini Kit (205113, Qiagen). RNA integrity was checked using the Agilent Bioanalyzer System. Dnase-treated RNA was treated for library preparation using the Truseq Stranded mRNA Sample Preparation Kit (Illumina, San Diego, CA), according to manufacturer’s instructions. An initial poly(A) RNA isolation step (included in the Illumina protocol) is performed on 10 ng of total RNA to keep only the polyadenylated RNA fraction and remove the ribosomal RNA. A fragmentation step is then performed on the enriched fraction, by divalent ions at high temperature. The fragmented RNA samples were randomly primed for reverse transcription, followed by second-strand synthesis to create double-stranded cDNA fragments. No end repair step was necessary. An adenine was added to the 3’-end and specific Illumina adapters were ligated. Ligation products were submitted to PCR amplification. The obtained oriented libraries were controlled by Bioanalyzer DNA1000 Chips (Agilent, # 5067-1504) and quantified by spectrofluorimetry (Quant-iT™ High-Sensitivity DNA Assay Kit, #Q33120, Invitrogen). Sequencing was performed on the Illumina Hiseq2500 platform to generate single-end 100 bp reads bearing strand specificity.

### Bioinformatic Analysis of the Genome-Wide Sequence Data

Bioinformatic analyses were performed using the RNA-seq pipeline from Sequana (44). Reads were cleaned of adapter sequences, and low-quality sequences were removed using cutadapt version 1.11. Only sequences ≥ 25 nucleotides (nt) in length were considered for further analysis. STAR version 2.5.0a, with default parameters, was used for alignment on the reference genome (*Chlorocebus sabaeus*, from Ensembl release 90). Genes were counted using featureCounts version 1.4.6-p3 (45) from Subreads package (parameters: -t gene, -g ID and -s 1).

Data were analyzed using R version 3.4.3 and the Bioconductor package DESeq2 version 1.18.1. Normalization and dispersion estimation were performed with DESeq2, using the default parameters, and statistical tests for differential expression were performed by applying the independent filtering algorithm. A generalized linear model, including the monkey identifier as a blocking factor, was used to test for the differential expression between the biological conditions. For each pairwise comparison, raw p values were adjusted for multiple testing according to the Benjamini and Hochberg (BH) procedure (46). Genes with an adjusted p value <0.05 were considered differentially expressed.

Analyses and visualization of GO terms associated with differentially expressed genes were performed using **ClueGO** (47). Both groups of genes (up- and downregulated, p value < 0.05) were used as dual input for GO and pathway annotation networks of the expressed genes and proteins pathway enrichment analysis. Each list was used to query the Kyoto Encyclopedia of Genes and Genomes (KEGG), GO-biological function database and Wiki pathways. ClueGo parameters were set as follows: Go Term Fusion selected; only display pathways with p values ≤ 0.05; GO tree interval, all levels; GO term minimum genes, 3; threshold of 4% of genes per pathway; and a kappa score of 0.42. GO terms are presented as nodes and clustered together based on the similarity of genes present in each term or pathway. The most significant term was chosen as a representative of the group (Benjamini-Hochberg correction).

### Statistical Analyses

Continuous variables were compared between groups throughout using non-parametric tests. Where three groups were compared, a Kruskal–Wallis test was used; pairwise comparisons were performed on all combinations of groups only if the overall test *p*-value was <0.05. To compare differences between two independent groups, the Mann-Whitney U test was used. Correlative analyses were performed using Spearman’s rank correlation. Correlation analyses were performed according to the Spearman coefficient of correlation.

Analyses were performed using GraphPad Prism (GraphPad Software, La Jolla, CA, USA) version 7.0 or R version 3.2.2.

## Results

### Tissue-Dependent Dynamics of CD32^+^CD4^+^ T Cells in Response to SIV Infection

To track and characterize CD32^+^CD4^+^ T cells within tissue compartments, we first performed a longitudinal measurement of CD32^+^CD4^+^ T cells in tissues before infection and during SIV infection in a natural host (AGM, N=17 animals) and a heterologous host (MAC, N=18 animals). AGM from the sabaeus species and cynomolgus MAC were respectively infected with the wild-type SIVagm.sab92018 and the SIVmac251 isolate (**Table S1**). We analyzed blood, LNs, spleen, ileum, jejunum and liver from uninfected, acutely infected and chronically infected animals (**Table S1**).

**Figure 1:**
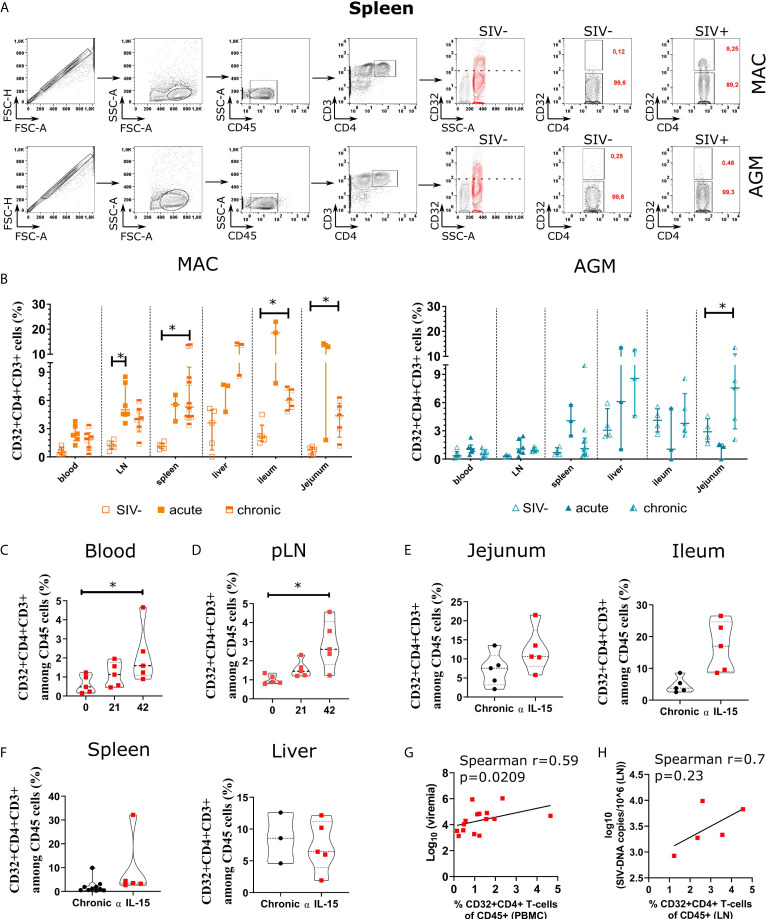
Quantification of CD4+ T cells expressing CD32 and/or viral RNA in tissues during pathogenic and natural host SIV infections. **(A)** Representative gating strategy for CD32+CD4+ T cells. A representative example from the spleen of a chronically infected cynomolgus macaque (MAC) (upper part), and a chronically infected AGM (lower part) are shown. The position of the CD32^+^ gate on CD4+ T cells was chosen according to the level of CD32 expression on myeloid cells (overlaid red population) in SIV-negative monkeys. In red is indicated the percentage of the gated population in both SIV- and SIV+ monkeys. **(B)** Graphs showing the frequency of CD32+CD4+ T cells in six tissues in SIV-uninfected, SIV acutely infected (day 9 p.i.) and SIV chronically infected MAC (orange) and AGM (blue). Values indicate the percentage of CD32+CD4+ T cells among total CD4+ T cells. Each individual monkey is represented by a square (MAC) or a triangle (AGM). The number of animals analyzed varied from three to six, depending on the compartment and time point studied. Time points or tissues with only three animals corresponding to liver and acute infection in gut were not included in the statistical comparisons. **(C, D)** Dynamics of CD32+CD4+CD3+ cells in blood and pLN of chronically SIV-infected AGM before, during and after anti-IL-15 administration. CD4+T cells were analyzed in blood and pLN before and at days 21 and 42 after initiation of anti-IL-15 treatment. The anti-IL-15 treatment of the chronically infected AGM (n=5 animals) has been previously reported (38). Violin plots showing the frequency of CD32+ CD4+T cells among CD45+ cells in blood **(C)** and pLN **(D)** in non-treated and anti-IL-15 treated chronically SIV-infected AGM. **(E, F)** Comparison of CD32+CD4+ T cells in tissues at necropsy, between chronically infected AGM treated or not with anti-IL-15. Violin plots show the distribution of CD32+CD4+CD3+ cells among CD45+ cells from chronically infected AGM (black) and anti-IL-15 treated chronically infected AGM (red) in the indicated tissue. **(G)** Frequencies of CD32+CD4+ T cells in PBMC of treated animals (day 42 post-anti-IL15) were plotted against viremia levels. **(H)** The frequencies of CD32+CD4+ T cells of treated animals (day 42 post-anti-IL15) were plotted against ca-viral DNA in LN. In **(B)**, statistical differences were assessed by ANOVA with Tukey adjustment for multiple comparisons. In **(C–F)**, a Kruskal-Wallis test was applied. Asterisks indicate p-values < 0.05. Each symbol represents a single animal.

To quantify the frequency of CD4^+^ T cells expressing CD32, we used for comparison CD32 expression levels on myeloid cells. The latter are well known to express high levels of CD32. We specifically defined a CD32 gate for each tissue and monkey using the level of expression of CD32 on blood monocytes as an internal positive control and fluorescence minus one (FMO) as a negative control (**Figure 1A** and **Figure S1**). The median of CD32^+^CD4^+^ T cells in blood, peripheral LN (pLN), spleen, liver, ileum, jejunum was 0.59%, 1.23%, 1.15%, 3.02%, 2.45% and 0.65%, respectively, in MAC and 0.53%, 0.32%, 0.79%, 3.62%, 4.11% and 3%, respectively, in AGM. After SIV infection, we observed an increase in CD32^+^CD4^+^ T cells in all lymphoid tissues (pLN, spleen, ileum and jejunum) of SIVmac-infected MAC, the average levels varying between 4.5% and 19% (**Figure 1B**). During SIVagm infection, a significant increase in CD32^+^CD4^+^ T cells was seen in the jejunum but not in LN (**Figure 1B**). Of note, the frequencies of CD32^+^CD4^+^ T cells were not increased in blood for neither MAC nor AGM, although we cannot exclude modest increases in some MAC. Altogether, these results showed that CD32^+^CD4^+^ T cells increased more in lymphoid tissues than in blood during SIV infection. CD32^+^CD4^+^ T cells were increased in both SLT and gut during SIVmac infection, but only in gut during chronic SIVagm infection.

### Increases in Viremia of SIV-Infected African Green Monkeys Are Associated With Increases in Tissue CD32^+^ CD4^+^ T Cell Frequencies

We wondered whether the lower increase of CD32^+^CD4^+^ T cells in SLT of AGM could be related to the strong control of SIV replication in SLT. To address this question, we investigated the levels of CD32^+^CD4^+^ T cells in NK-cell depleted AGMs infected with SIV. We and others have previously shown that anti-IL-15 treatment efficiently depletes NK cells *in vivo* in NHP (38, 48). Such NK cell depletion in chronically infected AGM increased both viremia as well as ca-viral DNA and RNA in SLT (38). We retrospectively monitored the frequency of CD32^+^CD4^+^ T cells from blood and LN of anti-IL15-treated and non-treated chronically infected AGM. There was an increase at day 42 post-anti-IL-15 of CD32^+^CD4^+^ T cells in blood and LN after NK cell depletion when compared to the frequency of these cells before anti IL-15 treatment (**Figures 1C, D**). We also compared the frequencies before and after NK cell depletion in other tissues (spleen, ileum, jejunum and in liver) (**Figures 1E, F**). There was a trend toward an increase in CD32^+^CD4^+^ T cells in the lymphoid tissues, such as ileum, but not in the liver (**Figure 1F**). The frequencies of CD32^+^CD4^+^ T cells in blood at day 42 post-anti-IL-15 correlated with viremia levels and there may be a trend toward correlation of CD32^+^CD4^+^ T cells with ca-viral DNA in the LN (**Figures 1G, H**). Taken together, these results show that CD32^+^CD4^+^ T cell frequencies increased in chronically infected AGMs in blood and LN, concomitant with an experimentally induced increase in viremia.

### CD32^+^CD4^+^ T Cells Are Enriched for SIV RNA in Secondary Lymphoid Tissues and Intestine

**Figure 2:**
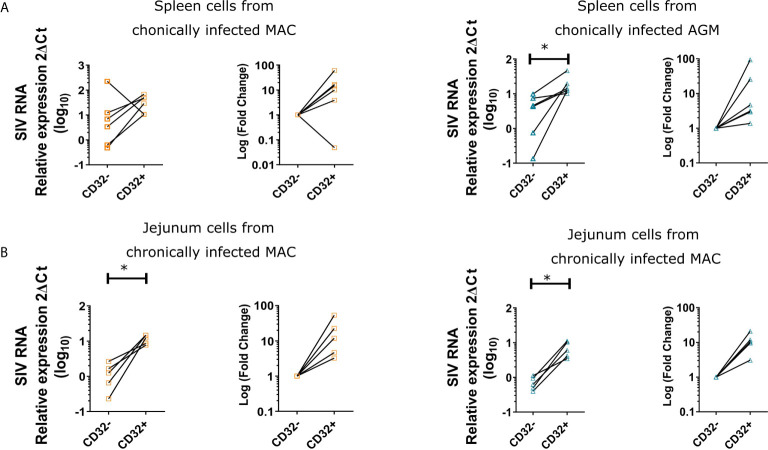
Quantification of ca-SIV RNA in CD32+ and CD32- CD4+ T cells. Cells were isolated from **(A)** spleen and **(B)** jejunum of chronically infected MAC and AGM. Graphs show the ca-SIV RNA amount relative to 18sRNA (left graph) and the fold change in ca-SIV RNA in CD32^+^ cells compared to CD32^-^ CD4^+^ T cells (right graph) for each species. The amount of RNA in CD32^-^ cells of each respective tissue was arbitrarily set to 1. Orange symbols refer to MAC and blue symbols to AGM. Each symbol represents a single animal. Statistical difference was assessed by a Mann-Whitney U-test. Asterisks indicate p-values < 0.05.

We next determined the level of viral replication in CD32^+^CD4^+^ T cells in tissues. We quantified ca-SIV RNA in CD32^+^CD4^+^ T cells from SLT (spleen) and intestinal mucosa (jejunum) of chronically infected MAC and AGM. The CD32^+^ and CD32^-^CD4^+^ T cell fractions were isolated by cell sorting. Cells were gated as Lin-CD45^+^CD3^+^CD4^+^CD32^+^/- (**Figure S1**). We detected viral RNA in each cellular fraction in all monkeys and all tissues analyzed. With the exception of the spleen of one chronically infected MAC, SIV RNA levels in the CD32^+^CD4^+^ T cell fraction were always higher compared to the CD32^-^CD4^+^ T cell fraction (**Figures 2A, B**). In the spleen, the fold change difference (median) of SIV ca-RNA was 12.4 and 3.9 in MAC and AGM, respectively, whereas in the jejunum it was 11.9 and 10.3 in MAC and AGM, respectively. Thus, CD32^+^CD4^+^T cells displayed higher levels of actively transcribed SIV RNA than CD32^-^CD4^+^T cells in SLT and gut in both SIVmac and SIVagm chronic infection. In SIVagm infection, the difference was less pronounced in SLT than in gut, consistent with lower viral replication levels in SLT of AGM.

### CD32^+^CD4^+^ T Cells in Secondary Lymphoid Tissues Express Markers of Activation and Preferential SIV Infection

**Figure 3:**
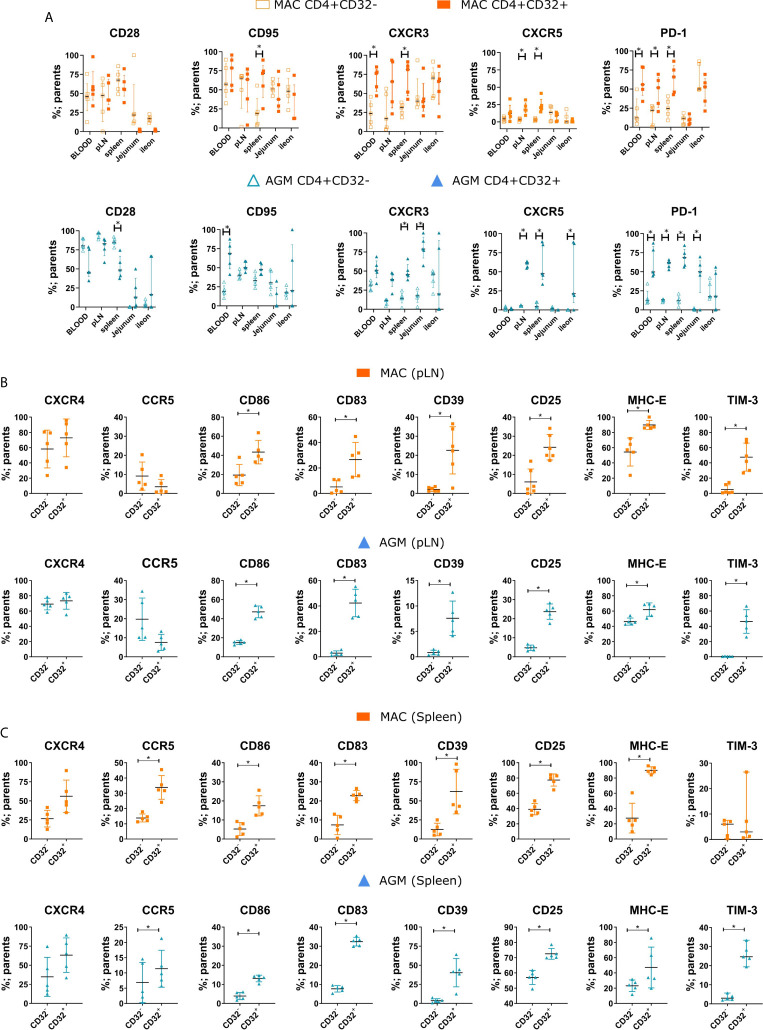
Phenotypic characterization of CD32+CD4+ T cells in tissues during SIV infection. **(A)** Frequencies of the indicated marker on CD32^-^ (empty symbols) and CD32+ (full symbols) for CD4+ T cells from different tissues during chronic SIV infection in MAC (orange) and AGM (blue). **(B, C)** Frequency of CD32^-^ and CD32+CD4+ T cells expressing a given marker in **(B)** peripheral LN (pLN) and **(C)** spleen during chronic SIV infection in MAC (orange) and AGM (blue). Each symbol represents a single animal (N=5 animals for each species). In **(A)**, two-way *ANOVA* with *Sidak* test for multiple comparisons was performed. In **(B, C)**, statistical difference was assessed by a Mann-Whitney U-test. Asterisks indicate p-values < 0.05.

We next assessed the phenotype of CD32^+^CD4^+^ T cells in SLT and gut. In a first step, we compared differentiation, homing, and exhaustion markers by multiparameter flow cytometry on CD32^+^ versus CD32^-^CD4^+^ T cells from blood, peripheral LN, spleen, ileum, and jejunum of chronically infected MAC and AGM. CD32^+^CD4^+^ T cells in blood and SLT were frequently PD-1^+^ compared to the respective CD32^-^ population in both species (**Figure 3A**). In SLT, CD32^+^CD4^+^ T cells also expressed more often CXCR5 and also more often CXCR3 in the spleen than CD32^-^CD4^+^ T cells in both MAC and AGM. In the gut, CD32^+^CD4^+^ T cells were also more often PD-1+, CXCR5^+^ and/or CXCR3^+^ than respective CD32^-^ cells in AGM. Given the common markers between TFH and CD32^+^CD4^+^ T cells, we analyzed if there is a correlation between these two cell populations. The TFH cell frequency correlated positively with the frequency CD32^+^CD4^+^ T cells in the pLN (**Figure S2**). Allover, CD32^+^CD4^+^ T cells expressed more frequently markers that were described to be often expressed on HIV-infected and/or HIV-reservoir cells in lymphoid tissues (i.e. PD-1 and CXCR5 in blood and SLT; CXCR3 in blood, SLT and mucosa) (8, 12, 14, 18–21).

We next turned our attention to the main co-receptors of SIV (CXCR4, CCR5), as well as markers known as evidence of activation (CD25), IFN-stimulation (CD83, CD86, MHC-E) and regulation (CD25, CD39, CD83, and TIM-3), with a focus on SLT (spleen and pLN) (**Figures 3B, C**). Strikingly, the differences between CD32^+^ and CD32^-^CD4^+^ T cells for all these 8 markers were generally the same for both species, indicating that the differences were not random. For instance, the frequencies of CD32^+^CD4^+^ T cells positive for CD25, CD39, CD83, CD86, and MHC-E were higher as for CD32^-^CD4^+^ T cells in both pLN and spleen of MAC and AGM. The CCR5^+^CD4^+^ T cells were also more frequent in the spleen within the CD32^+^ than the CD32- fraction in both species. Most of the CD32^+^CD4^+^ T cells also expressed CXCR4, although not to higher levels than the CD32^-^ cells because CXCR4 expression was already frequent among the latter. TIM-3 was most often increased in the CD32^+^ fraction of SLT as well (**Figure 3C**). All these markers were thus either similar or more frequent within the CD32^+^CD4^+^ T cells when compared to the CD32^-^ cells. CD25 is well known to be up-regulated upon CD4^+^ T cell activation and can be expressed at high levels on Treg. TIM-3 is known to be expressed by a subset of activated or exhausted CD4^+^ T cells and by polarized Th1 cells (49). CD39 is known to be found on activated CD4^+^ T cells with signs of metabolic stress (50, 51). There is increasing evidence that CD83 regulates CD4^+^ T cell development and peripheral activation (52–54). Altogether, these results suggest that CD32^+^CD4^+^ T cells are in a more activated state than CD32^-^CD4^+^T cells.

### CD32^+^CD4^+^CD3^+^ Cells Show Up-Regulated Expression of Genes Associated With B Cell Function

**Figure 4:**
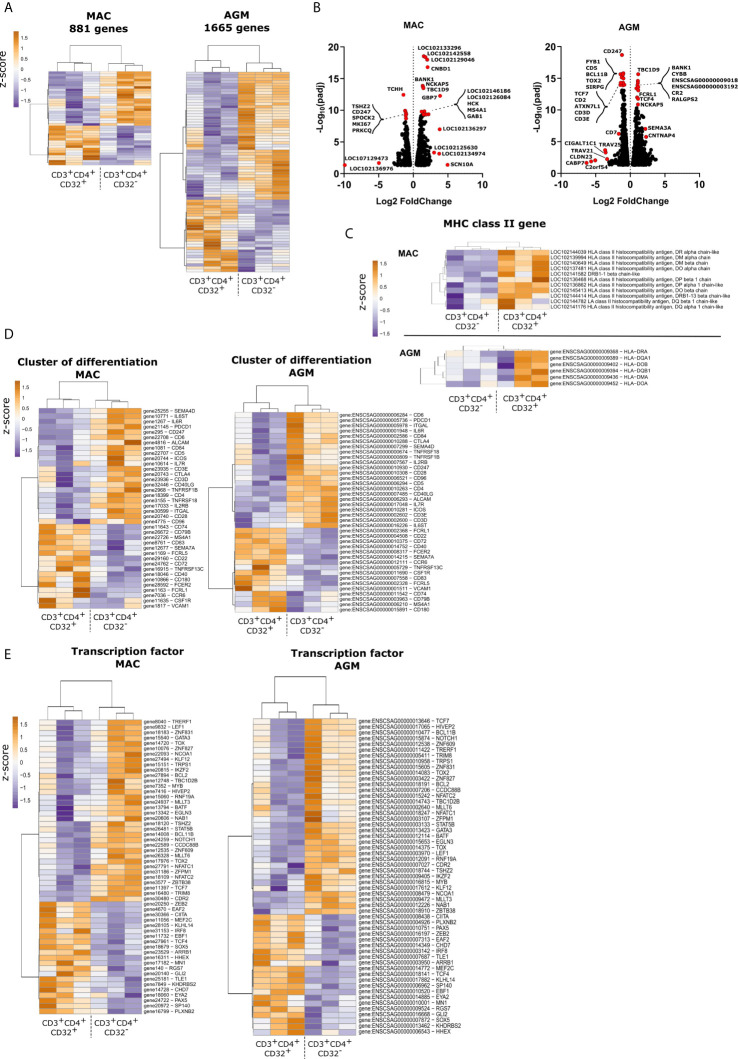
Genome-wide transcriptome analysis of CD4+ T cell subsets according to CD32 expression from spleen during pathogenic and non-pathogenic SIV infection. **(A)** Heatmaps of transcript signatures in CD32+ and CD32^-^CD4+ splenic T cells from chronically SIV-infected MAC and AGM. **(B)** Volcano plots of gene regulation between CD32+ and CD32^-^ CD4+ T cells. Red dots represent highly differentially regulated genes. Many of them are associated with the B cell lineage. **(C, E)** Heatmaps showing **(C)** MHC-II molecules **(D)** cluster of differentiation transcripts and **(E)** transcription factors mRNA levels on CD4+ T cell subsets according to CD32+ expression. In each panel, subsets were organized based on the overall similarity in gene expression patterns by an unsupervised hierarchical clustering algorithm of variable genes. A dendrogram, in which the pattern of branch length reflects the comparative difference in gene expression profiles between samples is shown. A p-value adjustment was performed to account for multiple comparisons and control the false positive rate to a chosen level. Transcriptome similarity between clusters of spleen sample was evaluated by the Euclidean distance and visualized *via* heatmap. Each row represents a variable gene among clusters, and each column represents one subset per monkey. Deep sequencing results were deposited in the Gene Expression Omnibus database; the accession number is GSE169736.

To investigate on a global scale, using a non-hypothesis-driven approach, the markers that distinguish CD32^+^ from CD32^-^CD4^+^ T cells in lymphoid tissues during SIV infection, we determined their genome-wide transcriptomic signature. CD32^+^CD4^+^ and CD32^-^CD4^+^ T cells were isolated as described above (**Figure 1A** and **Figure S1**). Cells were isolated from the spleen of three chronically SIVmac-infected MAC and three chronically SIVagm-infected AGM. CD32^+^ CD4^+^ and CD32^-^ CD4^+^ T cells clustered separately (**Figure 4A**) in both MAC and AGM. There were 881 and 1665 differentially expressed genes in CD32^+^ compared to CD32^-^CD4^+^ T cells for MAC and AGM, respectively (**Figure 4A** and **Table S2**). The genes that showed the highest expression in CD32^+^CD4^+^ T cells included T cell receptors (TRAV, CD3), but also many genes related to the B cell receptor rearrangement and other B cell markers, such as BANK1, a B cell transcription factor (**Figure 4B**). We also found upregulation of TBC1D9, a key regulator of TBK1. We did not found genes specifically related to the myeloid lineage, with one exception. Indeed, we observed an up-regulation of CD68 mRNA expression in CD32^+^ T cells of AGM (**Table S2**). However, any other classical monocyte-related gene, such as CD14, was not up-regulated. It has been shown that low levels of CD68 can be expressed in lymphoid cells such as CD19^+^ B lymphocytes and CD4^+^ T lymphocytes (55). Moreover, in vitro stimulation with T-cell mitogen or recombinant interleukin-2 (rIL-2) induced expression of CD68 antigen in activated CD4^+^ and CD8^+^ T lymphocytes (56). Thus, higher expression of CD68 in the CD32^+^ T cells is in agreement with CD32^+^ T cells harboring a higher state of activation than their CD32- counterpart.

To analyze the activation state of the cells, we assessed the level of major histocompatibility complex II (MHC II) receptor expression in CD32^+^CD4^+^ T cells. The level of HLA-DR transcripts in CD32^+^CD4^+^ T cells was higher compared with CD32^-^CD4^+^ T cells. Moreover, CD32^+^CD4^+^ T cells also expressed higher levels of MHC class II-transcripts encoding HLA-DP, -DQ, -DO, and -DM (**Figure 4C**).

We next analyzed in more detail all surface markers that were up- or down-regulated on the CD32^+^ cells compared to the CD32^-^ fraction (**Figure 4D**). Ninety-four genes encoding surface markers were differentially modulated in MAC and AGM together, out of which 38 genes were common between MAC and AGM (**Figure S3**). Many of these common genes that were up-regulated in the CD32^+^ cells are normally attributed to the B cell lineage, such as *MS4A1* (CD20), *CD22, CD40, CD72, CD79b, CD83 and CD74*, whereas transcripts encoding the CD4^+^ T cell lineage (i.e., *CD4, CD3e, CD3D, CD28, ICOS, CD5, IL7r, CTLA-4, and IL2RB*) were less expressed in CD32^+^ cells compared to CD32^-^CD4^+^ T cells (**Figure 4D** and **Figure S2**). Down-regulation of CD3 chains and CD4 mRNA is typical for activated CD4^+^ T cells (57–59). Both isoforms of CD32 were expressed, the CD32a isoform being less frequent than the CD32b isoform.

Cell types are also defined by transcription factors. We focused on the transcription factors that were commonly up- or down-regulated by CD32^+^CD4^+^ T cells in both MAC and AGM when compared to the CD32- fraction (**Figure 4E**). Fifty-five genes encoding transcription factors were differentially modulated between the CD32^+^ and CD32^-^ fractions in AGM and MAC (**Figure S4**). The CD32^+^ fraction expressed a high number of transcription factors linked to the B cell lineage, such as *PAX5*, whereas some key transcription factors for the T cell lineage were less expressed than in the CD32^-^CD4^+^ fraction (i.e. *BCL11B*, *GATA3*, *Notch1*).

We next attempted to understand the interferon-stimulated gene (ISG) expression profiles in the CD32^+^CD4^+^ T cells when compared to the CD32^-^CD4^+^ T cells. Many ISGs are known, and we included in particular those ISGs which are considered to have an antiviral function (60). The number of ISGs that were expressed to higher levels in CD32^+^ compared to CD32-CD4^+^ T cells was moderate: only 11 and 2 ISGs out of 41 ISGs analysed in, respectively, AGM and MAC (**Figure S5**). CD74 was the only ISG among those analysed that was commonly up-regulated in the CD32^+^ CD4^+^ T cells in both MAC and AGM.

To decipher the major functional pathways activated in the CD32^+^ and CD32- fractions, a Gene Ontology (GO) enrichment analysis was performed (**Figure S6A** and **Tables S3**, **S4**). Most of the pathways that were up-regulated both in MAC and AGM during SIV infection belonged to B cell receptor signaling pathways, while the pathways that were often down-regulated in both MAC and AGM in the CD32^+^CD4^+^ T cells as compared to the CD32^-^ fraction belonged to pathways involved in TCR signaling, T cell activation and differentiation (**Figure S6A** and **Tables S3**, **S4**). This was particularly true for CD32^+^ CD4^+^ T cells from MAC compared to AGM (**Figure S6B** and **Table S5**). Overall, this shows that the CD32^+^ cell fraction expressed many genes and pathways specific for B cells.

### CD32^+^CD4^+^CD3^+^ Cells Shared Phenotypic and Functional Aspects of B Cells

**Figure 5:**
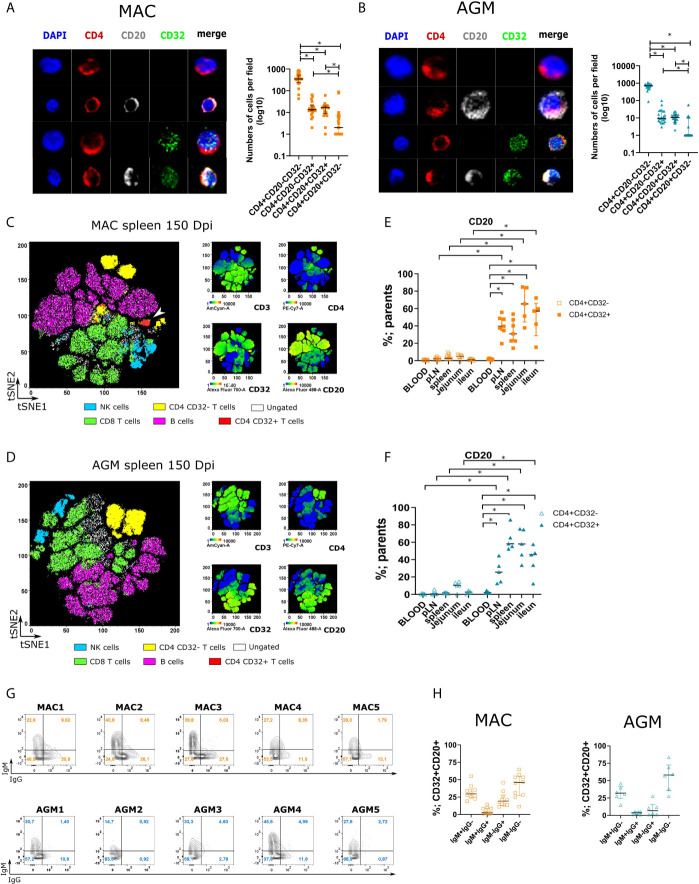
Analyses of CD32+CD4+CD3+ cells expressing CD20 in the spleen of SIV-infected animals. **(A, B)** Confocal images of CD4+ T cells according to CD4, CD20, and CD32 staining. Staining was performed on CD4+CD3+ cells isolated from chronically SIV-infected MAC **(A)** and AGM **(B)**. Graphs with cell numbers per field are shown on the right. The experiment was performed with samples from three monkeys per species and eight fields were counted per monkey. **(C, D)** viSNE map representing concatenated spleen cells from 6 MAC **(D)** and 6 AGM **(C)**. Cells were stained with 9 markers and measured by flow cytometry. viSNE analysis was performed on 60000 live CD45^+^ single cells per sample using all 8 surface markers. viSNE map shows concatenated flow cytometry standard files for all **(C)** MAC and **(D)** AGM samples. Overlay of 6 manually gated cell populations on viSNE plots, defined as: CD8+T cells (live, CD45+CD3+CD8+), NK cells (live, CD45+CD3-NKG2a+), B cells (live, CD45+CD3-CD20+), CD32+CD4+ T cells (live, CD45+CD3+CD4+ CD32+), CD32-CD4+ T cells (live, CD45+CD3+CD4+CD32-). Intensity of CD3, CD32, CD4, CD20, is shown for all samples, overlaid on the viSNE map. White arrows indicate CD32+CD4+ T cells. Cells not identified by such biaxial gating within CD45^+^ cells in the viSNE plots are shown in white. **(E, F)** Percentage of CD20+ cells within CD32+ CD4+ and CD32^-^ CD4+ according to their expression of IgM and IgG in MAC (orange) and AGM (blue). In Figure cells from distinct tissues of chronically infected **(E)** MAC and **(F)** AGM. **(G)** Frequency of IgM and IgG on CD4+CD20+CD32+ T cells in the spleen of chronically infected MAC (upper part) and AGM (lower part). **(H)** Distribution of CD32+CD20+CD4+ T cells according to their expression of IgM and IgG in MAC (orange) and AGM (blue). In **Figures 4A, B**, a Friedman test was applied. In **Figures 4C, D**, statistical differences were assessed by ANOVA with Tukey adjustment for multiple comparisons. Asterisks indicate p-values < 0.05.

The genome wide RNAseq thus revealed the expression of many genes associated with B cell function in the CD32^+^CD4^+^ T cells. This presence of a strong B cell signature within CD4^+^ T cells is generally unusual. We controlled for potential cell doublets. We coated freshly sorted spleen CD4^+^ T cells from chronically infected MAC and AGM and stained them for expression of CD4, CD20 and CD32. Confocal microscopy revealed the existence of cells co-stained positive for the three markers (**Figures 5A, B**). Most of the cells were CD3^+^CD4^+^CD32^-^CD20^-^ (>1.5 log more frequent than CD3^+^CD4^+^CD32^+^CD20^-^ cells). The CD20- and CD20^+^ frequencies among CD32^+^CD4^+^ T cells were comparable. Cells staining positive for CD20 were also observed within the CD32^-^ fraction but represented 1 log fewer cells than within CD32^+^ cells. This distribution was similar in MAC and AGM.

We then compared such CD32^+^ CD4^+^ T cells expressing CD20 to other immune cell subsets in the spleen. We performed a force-directed clustering analysis of total spleen cells isolated from chronically SIV-infected MAC and AGM based on the expression of eight markers. Individual flow cytometry standard files were concatenated into single flow cytometry standard files from which spatially distinct populations were obtained. To help identifying cell populations, traditional biaxial gating strategies based on surface markers were used as follows: CD4^+^ T cells (TCRβ^+^CD4^+^), CD8^+^ T cells (TCRβ^+^CD8^+^), B cells (CD20^+^), NK cells (NKG2A^+^). We overlaid immune cell populations identified by traditional gating strategies on viSNE plots and compared them to viSNE heat maps (**Figures 5C, D**). This allowed to easily identify spatially distinct populations corresponding to B cells, CD8^+^ T cells, CD32^-^ CD4^+^ T cells and NK cells in both species (**Figures 5C, D**). CD8^+^ T cells and NK cells showed some overlapping which is expected since some but not all NK cells express CD8 in tissues (61). The CD32^+^CD4^+^ cells (red) of MAC clustered in a unique, spatially distinct population expressing CD3, CD4, CD20 and CD32 markers. In AGM, the very low numbers of this population in spleen did not allow to define such clusters.

We next used the same technique to compare the frequency of CD32^+^CD4^+^ and CD32^-^CD4^+^ T cells expressing CD20 in distinct body compartments. We analyzed four tissues (spleen, LN, jejunum, ileum) and blood from MAC and AGM during chronic infection. In all lymphoid tissues analyzed, a high frequency of CD20^+^ cells was detected in the CD32^+^CD4^+^CD3^+^ population whereas a very low amount of CD32^-^CD4^+^CD3^+^ cells expressed this marker (**Figures 5E**, **F**). This differed from blood, where CD20 expression was also rare within CD32^+^CD4^+^T cells and not higher than for CD32^-^ cells. The frequencies of the CD20^+^CD32^+^CD4^+^CD3^+^ cells were thus much higher in SLT and gut than in blood.

To further confirm the sharing with B cell properties of the CD32^+^CD4^+^T cells, we analyzed functional markers of B cells in these cells. We determined the single and co-expression of IgM and IgG on CD32^+^CD20^+^CD4^+^ T cells from the spleen (**Figure 5G**). There was an absence of staining for both IgG and IgM antibodies on NK cells, CD3^+^CD4^-^ T cells, and CD4^+^CD32- T cells, whereas a high frequency of B cells as well as CD32^+^CD4^+^ T cells expressed those markers (**Figure S7**). The CD32^+^CD20^+^CD4^+^ T cells generally expressed either IgM or IgG, or none of them. Thus, >25% of the CD32^+^CD4^+^CD20^+^ T cells expressed IgM and not IgG, while some expressed IgG and no IgM (**Figure 5H**). About half of the CD32^+^CD4^+^ CD20^+^ T cells expressed neither IgG nor IgM.

## Discussion

The knowledge and understanding of the characteristics of CD4^+^ T cell subsets preferentially infected by HIV has considerably increased in the last years (62–64). Recent studies also provided information on major reservoir cells in tissues (12, 16, 65). Cell types are generally defined by specific markers. It is not unusual for some markers classically attributed to one type of cells to also be shared by a subfraction of other cell types For instance, mature CD8^+^ T cells, when activated, can co-express CD4 (66). Previous studies have identified CD4^+^ T cells expressing CD32a (25, 67–69). CD32a is an FcgR and is known to be primarily expressed on cell types such as myeloid cells, granulocytes, B cells and NK cells. Little is known about the function and biology of CD32^+^CD4^+^ T cells. Here, we analyzed CD32^+^CD4^+^ T cells in different tissues from two NHP models frequently used in biomedical research: AGM and cynomolgus MAC and investigated their dynamics in response to a viral infection. To this end, we studied these cells in healthy animals and during acute and chronic SIV infection. We performed tissue-level phenotypic and/or transcriptomic characterization at different stages of infection in lymphoid and non-lymphoid tissues. In addition, we characterized CD32^+^CD4^+^ T cells in the natural courses of SIV infection in AGM and compared these cells in tissues with well controlled (SLT) and not efficiently controlled (jejunum) SIV replication.

Our results show that CD32^+^CD4^+^ T cells were relatively rare in healthy NHP, similar to humans. The frequency of these cells strongly increased after SIV infection. The increase was compartment-specific, as the increase was very strong in lymphoid tissues (SLT, intestine) but not in blood, nor liver, during SIVmac infection. Some studies in humans also showed no significant differences in CD32^+^CD4^+^T cell frequencies in blood between HIV-1 negative, viremic and ART-treated individuals (31). Similar to MAC, an increase in CD32^+^CD4^+^T cells in the natural host was found in tissues and not in blood after SIV infection. However, the increases were pronounced only in the jejunum but not in LN. Natural hosts exhibit high viral replication in intestinal tissues, in contrast to SLT, where the viral replication is well controlled (37, 39, 70–73). Depletion of NK cells leads to loss of viral control in SLT in SIVagm infection (38). We show here that NK cell depleted SIVagm-infected AGM increase their CD32^+^CD4^+^T cells in SLT and that CD32^+^CD4^+^ T cell frequencies correlate with viremia levels. Altogether this shows that CD32^+^CD4^+^ T cells were preferentially increased in tissues with ongoing high-level viral replication.

Our genome-wide transcriptome analysis coupled with the phenotypic data indicated that CD32^+^CD4^+^ T cells were in a more activated state than CD32^-^CD4^+^T cells. In line with this, CD32^high^ CD4 T cells from blood of treated PLH have been described to express higher levels of HLA-DR and CD69 than other subsets (22). Other studies also suggested that CD32 marks highly activated/exhausted memory CD4^+^ T-cell subsets (74). The increased expression of CD32, which is an Fcγ receptor (FcγRIIa), could also make CD4^+^ T cells more susceptible to activation by IgG immune complexes.

CD32 expression might increase on CD4^+^ T cells as a direct consequence of the virus, either through viral infection and/or antigenic stimulation (25). Indeed, stimulation of CD4^+^ T cells with anti-CD3/CD28 antibodies has been described to induce CD32 co-expression (75). The CD32^+^CD4^+^ T cell profiles in SLT and gut upon SIVagm infection suggest such a direct effect of the virus. However, the inflammatory environment induced by chronic viral replication might also favor the emergence of such cells. External factors, such as IL-2, IL-7 and PHA, have been shown to induce CD32 expression on CD4^+^ T cells *in vitro* (75). The experimental NK cell depletion in AGM was induced by anti-IL-15. While most NK cell populations collapse in the absence of IL-15, effector memory CD4^+^ T cells can be maintained in the face of IL-15 inhibition by the activity of other homeostatic regulators, such as IL-7 (48). Interestingly IL-7 is known to maintain B cell potential in common lymphoid progenitors (76). Anti-IL15 treatment was associated with an increase in CD32^+^CD4^+^ T cells. Therefore, it cannot be excluded that the anti-IL-15 treatment, by inducing IL-7 as a homeostatic response, promoted the appearance of CD32^+^CD4^+^ T cells through a bystander effect in anti-IL-15 treated animals. IL-7 can also be increased in HIV-1 and SIVmac infection as a consequence of CD4^+^ T cell depletion, as shown during primary infection in the blood and intestine (77–79). Thus, the increase in CD32^+^ CD4^+^ T cells might be a mixture of factors directly and indirectly related to HIV/SIV replication.

The higher activation state of CD32^+^CD4^+^ T cells may explain the higher frequency of HIV RNA transcription that we observed compared to the other CD4^+^ T cells. The CD32 in splenic CD4^+^ T cells marked highly transcriptionally active CD4 T cells. Our data are in agreement with other studies reporting transcriptionally active virus in these cells in blood (29, 31, 80), and with data on LN from HIV-infected individuals showing HIV RNA in CD32^+^ cells inside B cell follicles (31, 81, 82). We also observed an up-regulation of CD74, an ISG known to be up-regulated in activated infected cells (83, 84) and known to be involved in the formation and transport of MHC class II peptide complexes for the generation of CD4^+^ T cell responses. The viral DNA has not been measured here and future studies will need to address if CD32^+^CD4 T cells from tissues contain more SIV DNA than CD32-CD4^+^ T cells and if the frequency CD32^+^CD4 T cells in tissues correlates with ca-SIV DNA. Altogether, CD32^+^CD4^+^ T cells displayed higher levels of actively transcribed SIV RNA than CD32^-^CD4^+^ T cells in SLT and gut during both SIVmac and SIVagm chronic infection.

We also show that CD32^+^CD4^+^ T cells from the spleen expressed more often CCR5 than the other CD4^+^ T cells. CD32^high^CD4 T cells from the blood of treated PLH have indeed been described in other studies to express higher levels of HIV co-receptor expression than other subsets (22). Our data are therefore compatible with a phenotype of these CD32^+^ CD4^+^ T cells being particularly susceptible to HIV/SIV infection.

The CD32^+^CD4^+^ T cells often expressed PD-1 and CXCR5. This could have several explanations. It has been suggested that they resemble T_FH_ cells (81). We show here that approximately 90% of CD32^+^CD4^+^ T cells from LN were MHC-E positive. We have previously reported that T_FH_ cells in SLT express MHC-E more frequently than any other CD4^+^ T cell subset (85). This is an additional argument for CD32^+^ T cells exhibiting T_FH_ cell characteristics. However, it could also be that CXCR5 and PD-1 expression is associated with the B cell phenotypical characteristics of these cells. In the lymphoid tissues, but not in the blood, about half of the CD32^+^ CD4^+^ T cells in the SIV-infected NHPs also expressed the B lymphocyte antigen CD20 at the cell surface. The observation that CD20^+^CD4^+^CXCR4^+^ T cells can be infected with HIV-1 *in vitro* was reported many years ago (68). More recently, Serra-Peinado et al. described that CD20^+^CD4^+^ T cells from blood and LN of patients on antiretroviral therapy (ART) were significantly enriched in HIV transcripts (23). Our study supports that CD20 can be expressed on target cells of HIV *in vivo*. Here, we provide an in-depth analysis of the molecular properties of CD32^+^CD20^+^CD4^+^ T cells. We combined distinct genomic, immunological and imaging methods to confirm the existence of this peculiar cellular subset. The high content of mRNA encoding transcription factors and surface markers usually attributed to the B cell lineage in the transcriptome analysis of CD32^+^ CD4^+^ T cells made the presence of contaminating cells unlikely. Furthermore, mRNA expression excluded the possibility of an acquisition of surface molecules attributed to the B cell lineage by trogocytosis as previously proposed (26, 29). Previous reports also have proposed that T-cell–B-cell conjugates may be the source of CD32 and CD4 co-expression (26). The imaging analyzes performed here on total CD4 T isolated from infected monkeys confirmed the existence of cells co-expressing CD32, CD4, and CD20. However, it is difficult to completely exclude the possibility of doublets contributing to this signal in some of the FACS based experiments. Of note, several reports have shown that B cells exclusively express the CD32b isoform (29, 86). Here, we found that the CD32 a isoform was present in all sorted CD32^+^ CD4^+^ T cell fractions of SIV-infected monkeys. This also demonstrates that CD32 co-expression on the CD4^+^ T cells cannot be solely explained by eventual doublets. Our results are in agreement with other studies showing that many of the CD32^high^ CD4^+^ T cells from HIV-1^+^ patients, and from healthy donors, co-express multiple B cell markers (22, 23, 29). Of note, we show here that CD20 expression was frequent on CD32^+^CD4^+^ T cells from gut and SLT but not for blood CD32^+^CD4^+^ T cells during SIV infection, underlining tissue-dependent distribution.

In the present study, genome-wide transcriptome analysis revealed that the CD32^+^CD4^+^ T cells detected here expressed low levels of *GATA-3*, *BCL11b* and *Notch1*, but high levels of *IRF8* compared with the CD32^-^CD4^+^ cell fraction. Previous studies showed that IRF8 could play a role in the earliest stages of B-cell development (87). Notch induces T cell factor 1 (Tcf1), which is the first T cell-specific protein in the thymus, leading to the activation of two major target genes, *Gata3* and *Bcl11b*. GATA-3 is known to critically suppress a latent B-cell potential in pro–T cells (88–90). BCL11B also supports the maintenance of T-cell fate by continuously suppressing epigenetic changes in the B-lineage-specific gene program (91). Thus, the expression pattern indicates the presence of transcription factors down-regulating T cell pathways in favor of the B cell lineage. Overall, the differential expression of checkpoint molecules of the T and B cell lineage fates suggests the presence of regulatory mechanisms of the early T and B cell differentiation pathways in this CD32^+^CD4^+^ T cell subset.

Recent data provided strong evidence that compartmentalization of T and other cells, such as B cells, is not absolute: violators of this paradigm can indeed be generated under specific conditions (92–95). CD20-expressing T cell populations have been found in healthy individuals as well as in a variety of non-malignant disease states (92, 94, 96–100). CD20^+^ inflammatory T-cells have been described in blood and brain of multiple sclerosis patients (94, 101, 102). Murayama and colleagues reported significant increase of CD3^+^CD4^+^CD20^+^ T cells in lymph nodes and not in blood during SIV infection in MAC at the stage of lymphadenopathy (103).

Thus, under conditions where the tissue microenvironment is modified, such as during chronic HIV/SIV infection in lymphoid tissues, modifications in cell differentiation could be favored.

Whether CD32^+^CD20^+^ T cells have a specific function or are only a byproduct remains to be clarified. In the case of HIV/SIV infection, the expansion of such cells might be harmful as they seem highly susceptible to infection. The fact that they increase already in acute infection, as shown here, raises the question of their contribution to the viral reservoir that is established early on after HIV/SIV infection. It is not excluded though, that these CD32^+^CD20^+^CD4^+^ T cells also have a beneficial role in viral or immunological diseases. This question needs to be addressed in future studies.

Altogether, we show that CD32^+^CD4^+^ T cells had an activated profile, more frequently expressed markers associated with HIV infected and/or reservoir cells (CCR5, PD-1, CXCR5, CXCR3) and displayed higher levels of actively transcribed SIV RNA than CD32^-^CD4^+^T cells. We show that CD4^+^ T cells expressing CD32 were rare in healthy animals but strongly increased after SIV infection in tissues exhibiting higher replication and immune activation. These CD32^+^CD4^+^ T cells in tissues also often expressed B cell markers. Genome-wide transcriptome revealed a coordinated regulation of T and B cell fate checkpoint molecules. Our results suggest that the tissue microenvironment associated with viral replication in gut and SLT drives the differentiation of a functionally not well-described subpopulation of activated CD4^+^ T cells with enhanced susceptibility to HIV infection in lymphoid tissues.

## Data Availability Statement

Deep sequencing results have been deposited in the Gene Expression Omnibus database; the accession number is GSE169736. The authors declare that all other data supporting the findings of this study are available within the article and its Supplementary Information files or are available from the authors upon request.

## Ethics Statement

The animal study was reviewed and approved by Animal experimental protocols were approved by the Ethical Committee of Animal Experimentation (CETEA-DSV, IDF, France) (Notification 12-098 and A17-044). The pVISCONTI study was approved and accredited under statement number A15-035 by the ethics committee “Comité d’Ethique en Expérimentation Animale du CEA”, registered and authorized under Number 2453-2015102713323361v2 by the French Ministry of Education and Research.

## Author Contributions

NH and MM-T designed the study. NH designed the experiments. NH, PR, CyP, BJ, ET, RL and HV performed experiments. NH, HV, EK and RL performed statistical and bio-informatic analyses. NH, PR, Cyp, A-SB, AS-C, HM, BJ and MM-T analyzed the data. VC, Cap, RG and BJ coordinated the animal studies. NH and MM-T wrote the manuscript and all co-authors reviewed it. All authors contributed to the article and approved the submitted version.

## Funding

For sequencing and analysis support, we thank the Biomics Platform, C2RT, Institut Pasteur, Paris, France, supported by France Génomique (ANR-10-INBS-09-09) and IBISA. NH was supported by the Fondation Jacquelin Beytout and Institut Pasteur. PR was recipient of a PhD fellowship from the University Paris Diderot, Sorbonne Paris Cité and also supported by the NIH (R01AI143457). CP was the recipient of a PhD fellowship from the ANRS. HM received core grants from the Institut Pasteur, the INSERM and the Milieu Intérieur Program (ANR-10-LABX-69-01). We would like to acknowledge funding support from ANRS, MSDAvenir and Institut Pasteur to MM-T and AS-C. We gratefully acknowledge the support to IDMIT from the French government: Investments for the Future program for infrastructures (PIA) through the ANR-11-INBS-0008 grant as well as from the PIA grant ANR-10-EQPX-02-01 to the FlowCyTech facility at IDMIT.

## Conflict of Interest

The authors declare that the research was conducted in the absence of any commercial or financial relationships that could be construed as a potential conflict of interest.

## Supplementary Material

Supplementary Figure 1 Gating strategy used to identify and isolate CD32+CD4+ T cells in tissues from uninfected and SIV-infected AGM and MAC. **(A)** Example of the gating strategy used to isolate CD32+CD4+ T cells from healthy MAC. The red square highlights the dot plot showing CD3+CD20+ cells **(B)** Example of dot plot showing CD3+CD20+ cells obtained during FACS sorting from chronically infected MAC and healthy or chronically infected AGM.

Supplementary Figure 2 Gating strategy used to identify TFH cells in pLN from uninfected and SIV-infected MAC and AGM. Graph showing Spearman’s correlation between TFH and CD32+CD4+ T cell frequencies. Each dot represents an individual from acutely (day 9 Pi; purple dot) or chronically infected animals (black dot). The Spearman correlation is shown, and r and p values are indicated in each graph.

Supplementary Figure 3 Venn diagrams generated by the intersection of transcripts encoding clusters of differentiation (CD) with a p-adj <0.05 when CD32+ CD4+ are compared with CD32-CD4+ fraction in the spleen of chronically SIV-infected MAC (orange circle) and AGM (blue circle). Up-regulated clusters of differentiation (CD) are shown in the top of the figure and down-regulated clusters of differentiation (CD) are shown in the bottom part. The number of differentially regulated genes is indicated in each circle, and the list of each gene is below the circles.

Supplementary Figure 4 Venn diagrams generated by the intersection of transcripts encoding transcription factors (TFs) with a p-adj <0.05 when CD32+CD4+ T cells are compared with CD32- CD4+ T cell fraction isolated from the spleen of chronically SIV-infected MAC (orange circle) and AGM (blue circle). Up-regulated TFs are shown in the top of the figure and down-regulated TFs are shown in the bottom parts. The number of genes is indicated in each circle, and the list of each gene is provided below.

Supplementary Figure 5 Interferon-stimulated-genes (ISGs) differentially expressed within CD32+CD4+ T cells. The Venn diagram was generated by the intersection of all differentially expressed transcripts with a p-adj <0.05 found in CD32+CD4+ T cells from the spleen of chronically SIV-infected MAC (red circle), AGM (green circle) and ISG genes (blue circle). The ISG genes are as reported by Schoggins and Rice (60). In AGM, Eleven ISG genes were found to be differentially expressed within CD32+CD4+ T cells (i.e, OAS, GBP1, OAS2, SLC15A3, MAP3K14, ZC3HAV1, DDX60, MOV10, IFI6, IFI44L, DDX58), whereas two ISG genes were found to be differentially expressed in MAC (i.e, SSBP3, ISG20). Only CD74 is found to be differentially expressed within CD32+CD4+ T cells in both MAC and AGM.

Supplementary Figure 6
**(A)** Pie chart showing the up- and down-regulated signaling pathways in CD32+CD4+ T cells isolated from the spleen of chronically SIV-infected MAC and AGM. **(B)** Pie chart showing the up- and down-regulated common signaling pathways in CD32+CD4+ T cells isolated from the spleen of chronically SIV-infected MAC and AGM. Common signaling pathways between AGM and MAC were defined by using common up- and down-regulated transcripts in the CD32+CD4+ T cell subsets when compared to the CD32-CD4+ T cells.

Supplementary Figure 7 Gating strategy used to determine the frequency of CD32+CD4+ T cells expressing IgG and/or IgM from the spleen of chronically SIV-infected AGM **(A)** and MAC **(B)**.

Supplementary Table 1 Table showing the characteristics of the monkeys used in this study.

Supplementary Table 2 List of genes up- and down-regulated in CD32+CD4+ T cells when compared to CD32-CD4+T cells.

Supplementary Table 3 Pathway up- and down-regulated in CD32+CD4+ T cells when compared to CD32-CD4+T cells in chronically infected MAC.

Supplementary Table 4 Pathway up- and down-regulated in CD32+CD4+ T cells when compared to CD32-CD4+T cells in chronically infected AGM.

Supplementary Table 5 Common pathways up- or down-regulated in AGM and MAC between CD32+CD4+ T cells when compared to CD32-CD4+T cells. Pathway analysis was performed using all common transcripts with a p-adj <0.05 found in CD32+CD4+ T cells from the spleen of chronically SIV-infected MAC and AGM.
