# Supplementary material for: CD32+CD4+ T Cells Sharing B Cell Properties Increase With Simian Immunodeficiency Virus Replication in Lymphoid Tissues
Source: Front Immunol. 2021 Jun 16;12:695148. doi: 10.3389/fimmu.2021.695148 (PMC8242952; doi:10.3389/fimmu.2021.695148)
Supplement: Supplementary Figure 1 — Gating strategy used to identify and isolate CD32+CD4+ T cells in tissues from uninfected and SIV-infected AGM and MAC. (A) Example of the gating strategy used to isolate CD32+CD4+ T cells from healthy MAC. The red square highlights the dot plot showing CD3+CD20+ cells (B) Example of dot plot showing CD3+CD20+ cells obtained during FACS sorting from chronically infected MAC and healthy or chronically infected AGM. [file DataSheet_1.pdf]

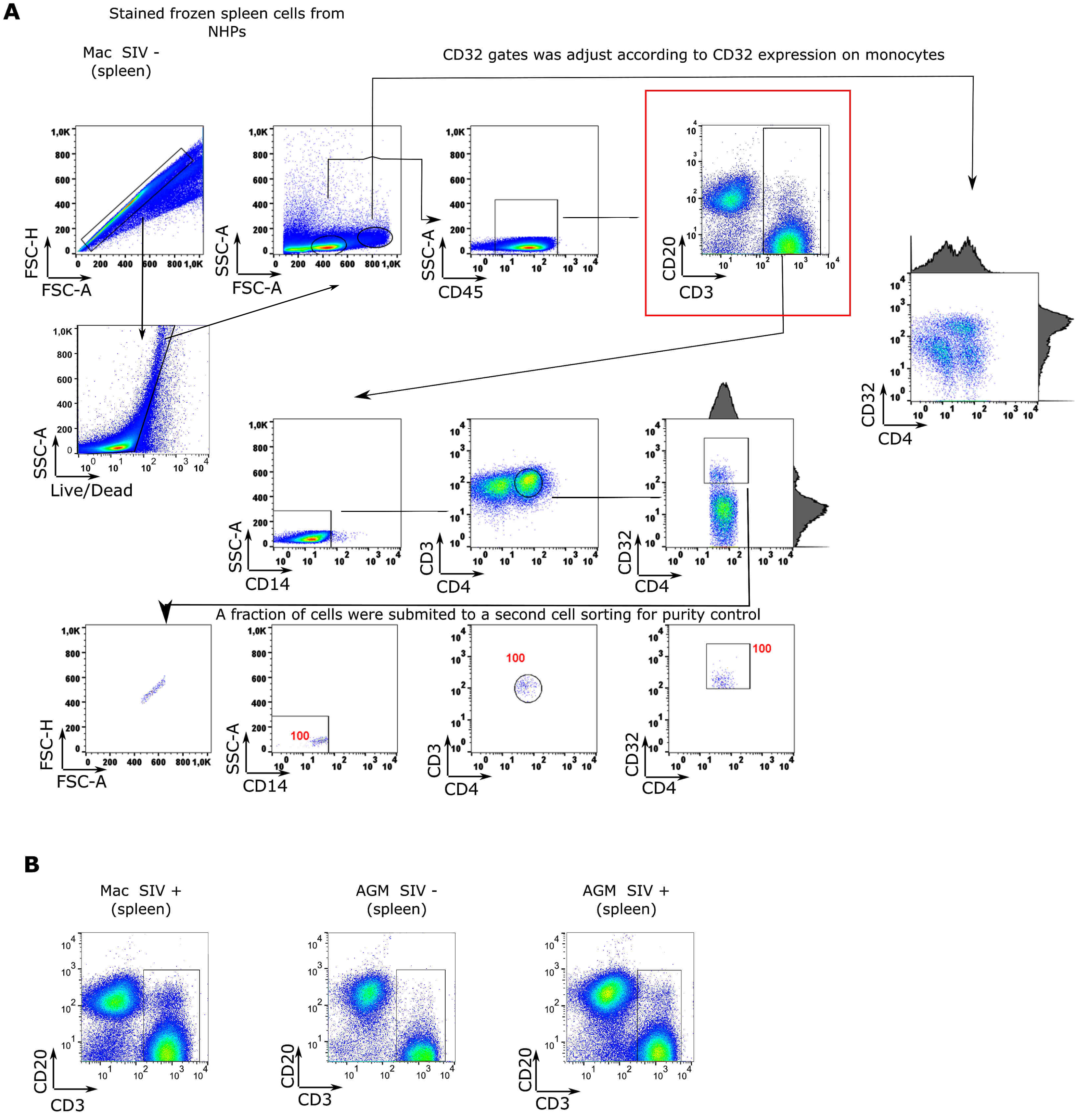
A
Stained frozen spleen cells from NHPs
Mac SIV -
(spleen)
CD32 gates was adjust according to CD32 expression on monocytes
FSC-H
FSC-A
SSC-A
FSC-A
SSC-A
CD45
CD20
CD3
CD32
CD4
SSC-A
Live/Dead
SSC-A
CD14
CD3
CD4
CD32
CD4
A fraction of cells were submited to a second cell sorting for purity control
FSC-H
FSC-A
100
SSC-A
CD14
100
CD3
CD4
100
CD32
CD4
B
Mac SIV +
(spleen)
AGM SIV -
(spleen)
AGM SIV +
(spleen)
CD20
CD3
CD20
CD3
CD20
CD3

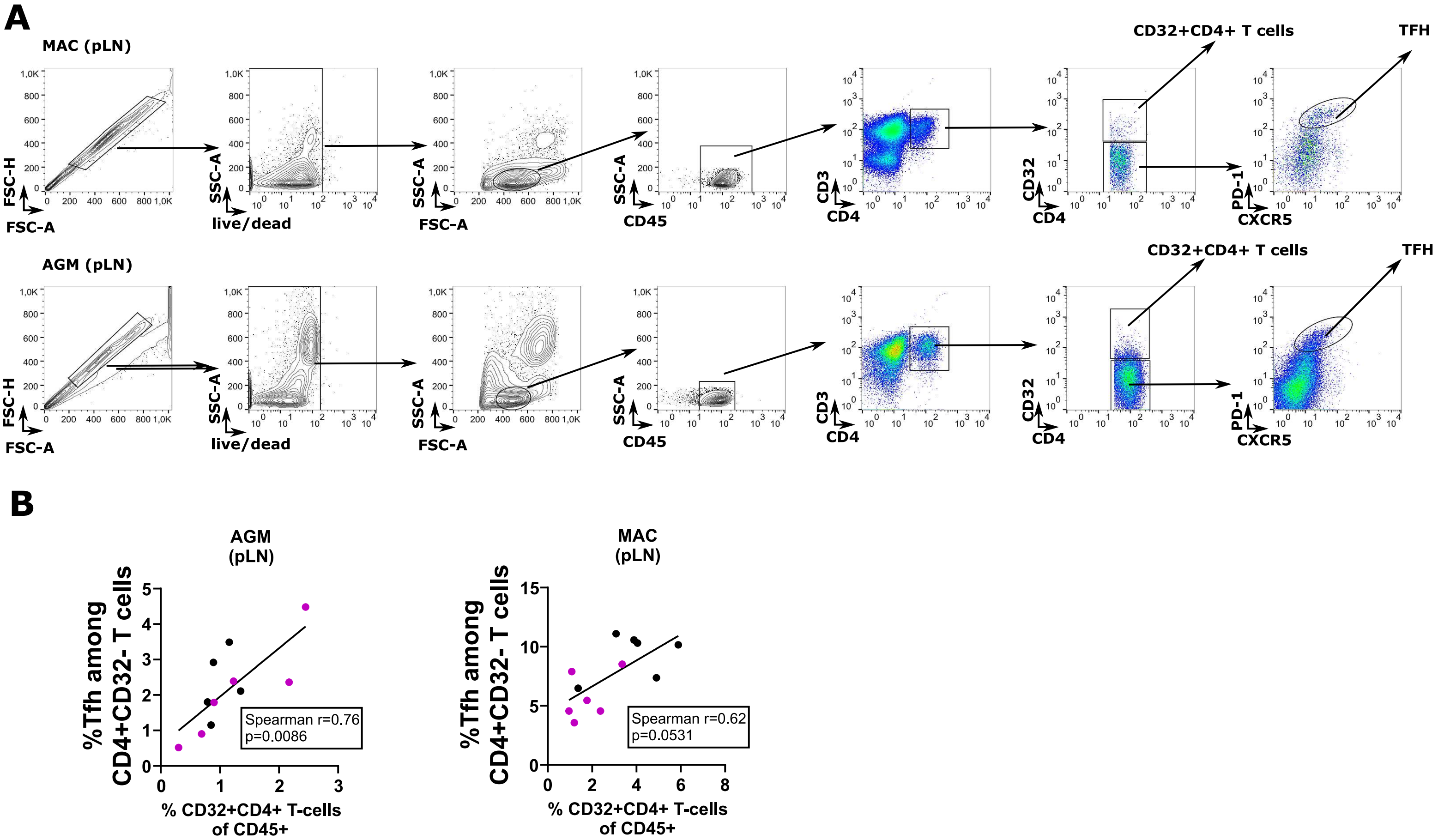
A
MAC (pLN)
CD32+CD4+ T cells
TFH
FSC-H
FSC-A
SSC-A
live/dead
CD45
CD3
CD4
CD32
PD-1
CXCR5
AGM (pLN)
B
AGM
(pLN)
MAC
(pLN)
%Tfh among
CD4+CD32- T cells
% CD32+CD4+ T-cells
of CD45+
Spearman r=0.76
p=0.0086
Spearman r=0.62
p=0.0531

CD32+CD4+ T cells versus CD32-CD4+ T cells
Up regulated genes coding for cluster of differenciation

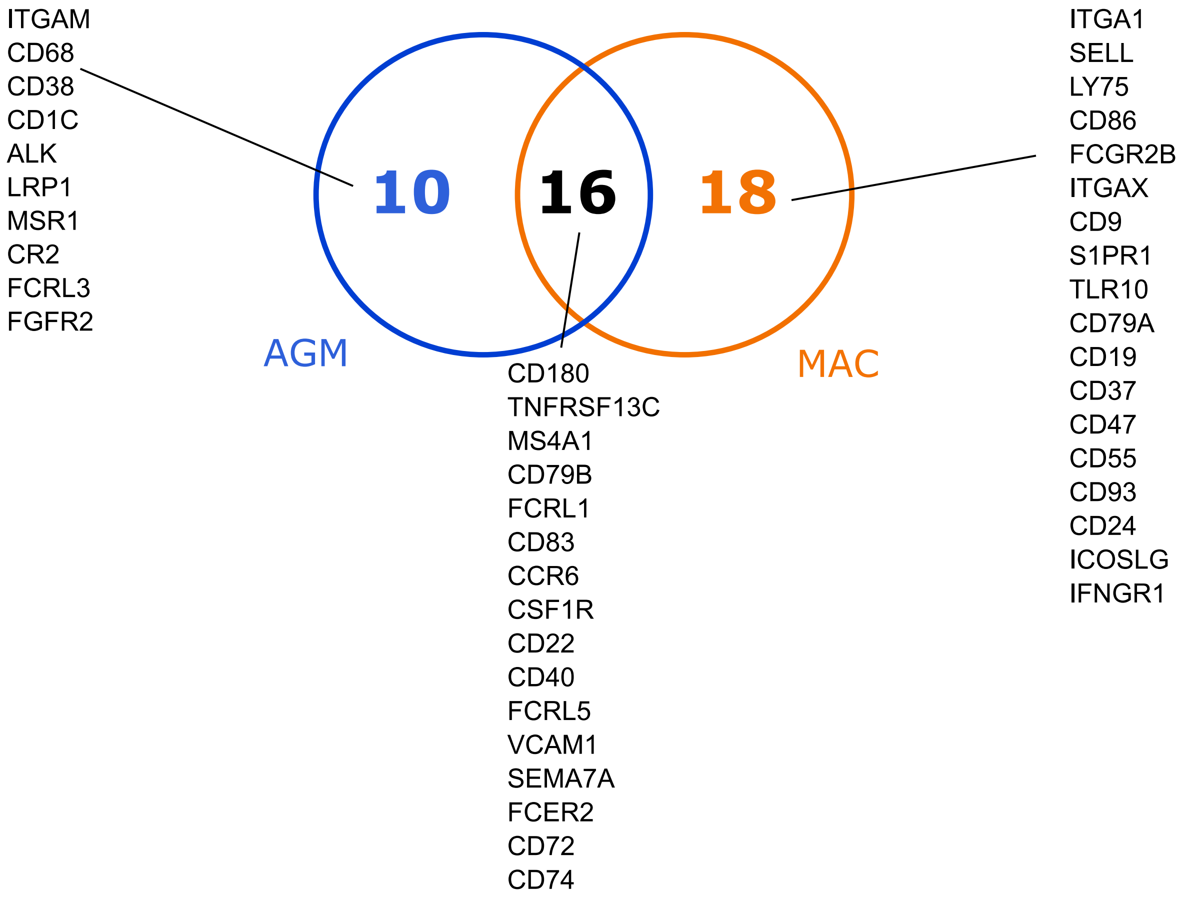

CD32+CD4+ T cells versus CD32-CD4+ T cells
down regulated genes coding for cluster of differentiation

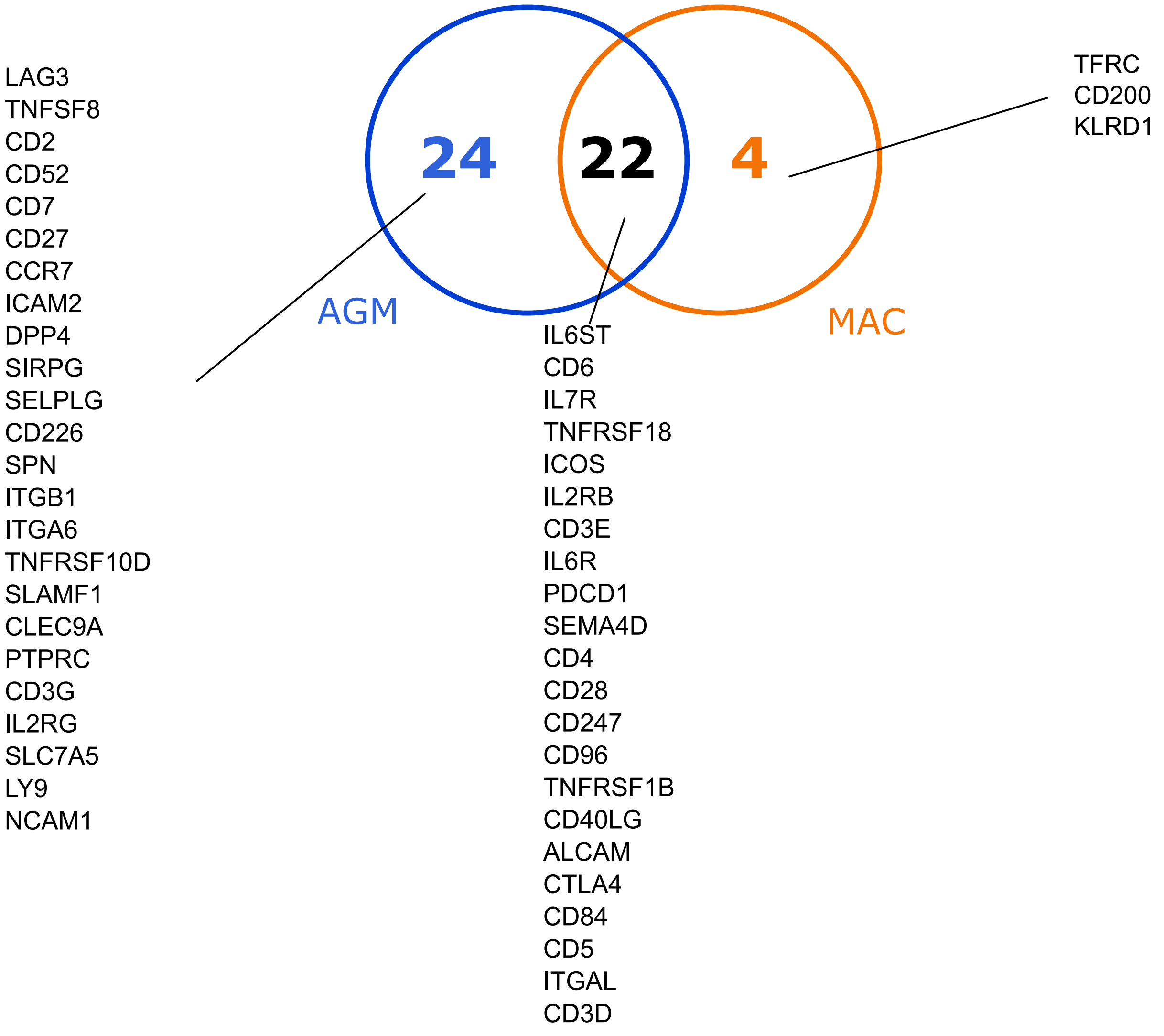

CD32+CD4+ T cells versus CD32-CD4+ T cells
Up regulated genes coding for transcription factors

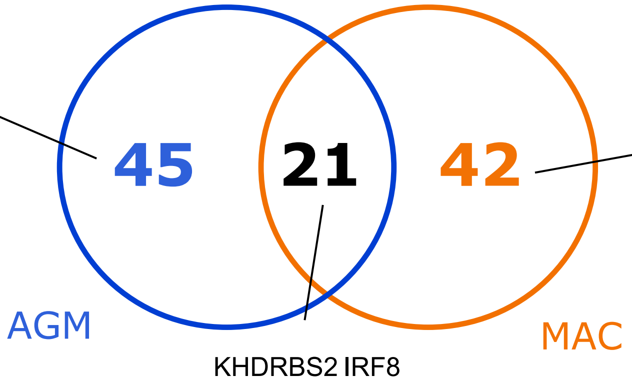

AGM (45):

MAML3	DZIP1L
DACH2	HLA-DQB1
DEPTOR	PCSK6
FAM171B	THRB
MYRF	PCBP3
ZDHHC21	MEIS1
MEIS2	ESRRG
ZSCAN18	GRHL2
ZNF704	BNC2
EYA1	MET
PAX8	AMOT
ELL3	ZMAT4
PKHD1L1	PREX2
ZFHX4	ZNF215
MITF	PLXNA2
ZNF385D	NR1H4
EBF3	NOTCH2
TFEB	SORBS2
SPI1	FBN1
RAPGEF5	SLC4A10
LIMD1	ETV5
RFX4	ZNF318
ZNF423	

Common (21):

KHDRBS2	IRF8
RGS7	ZEB2
PAX5	EAF2
CIITA	TLE1
CHD7	EBF1
GLI2	EYA2
SOX5	MN1
KLHL14	MEF2C
TCF4	HHEX
SP140	ARRB1
PLXNB2	

MAC (42):

BMP4	LDB2
RGS9	HDAC9
GPR155	LITAF
POU2AF1	S100A1
BTG2	MNDA
MEFV	XBP1
CREB3L2	RAI14
ZNF385A	PIKFYVE
RNF144A	AEBP1
PDLIM1	NRG1
RARB	PLEK
NR1D2	ZNF395
TP73	AFF3
AFF1	CUX2
IRF5	ABCA4
ZNF536	KIAA0040
DENND4A	BCL11A
ADRB2	ESR1
JUP	L3MBTL4
L3MBTL3	ENC1
SETBP1	RUFY3

CD32+CD4+ T cells versus CD32-CD4+ T cells
down regulated genes coding for transcription factors

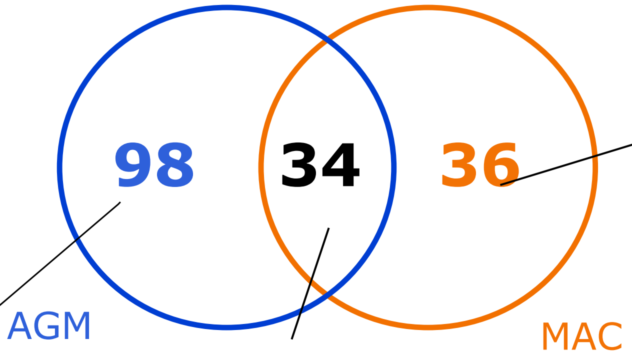

AGM (98):

PPARD	APP	RFX8
ARID4B	HTATSF1	RORA
ZC3HAV1	ZC3H7A	PIAS2
CHMP3	SPEN	ZNF57
YEATS2	HSF1	GATAD2A
MKL1	NFATC2IP	TECPR1
JUNB	LDB1	HDAC2
CNBP	FOXK1	ATF1
ZC3H13	PHF21A	OPTN
SUFU	KMT2A	ETS1
RBL2	NCOR2	TCF12
TSPYL2	RGS14	KLHL3
NCOA7	RGCC	SYNE1
CREBRF	PKN1	SRSF3
JUND	ARHGAP35	FLNA
ING4	TOPORS	MSL3
SCAPER	PLXND1	RNF125
SUB1	ZFP36L2	ZNF292
TGFB1	ASH1L	BAZ1B
ETV6	TSC22D4	MTA3
PPP1R13E	FOXM1	EPS15
PBXIP1	SEC14L2	H1FX
EPC1	ANKZF1	NCOA6
ZNF512B	RBM15B	STAT1
NFIC	PSIP1	RIN3
CHD6	MBNL1	RUNX1
ZSCAN26	ZSCAN21	ID2
SSH2	DNAJC1	HMGB2
STAT4	THRA	SATB1
JUN	TFCP2	SSBP2
TXK	BPTF	KAT6B
AKNA	SCML4	KAT2B
TCF20	MAML2	

Common (34):

NAB1	ZNF609
RNF19A	ZNF827
LEF1	NCOA1
BCL2	NOTCH1
STAT5B	HIVEP2
TBC1D2B	NFATC1
TRERF1	MLLT6
CCDC88B	EGLN3
IKZF2	TRIM8
TRPS1	BATF
BCL11B	TSHZ2
MLLT3	NFATC2
GATA3	TOX2
ZFPM1	CDR2
MYB	ZBTB38
KLF12	ZNF831
TCF7	TOX

MAC (36):

OTUD7B	NCALD
HPCAL1	CHD3
YY1	KEAP1
NFKBIA	BRCA2
CARHSP1	ATXN1
APBB2	NFIA
HIVEP1	MKL2
APOL2	ZGPAT
ZKSCAN5	BTBD11
BAZ2B	SSBP3
MGAT5	HDAC4
CHD8	PACS2
ZSCAN25	PHF19
HMGN5	MAF
GFI1	ZNF16
DNMT3A	CPSF4
ZNF101	CENPF
DEDD2	TRIM13

A

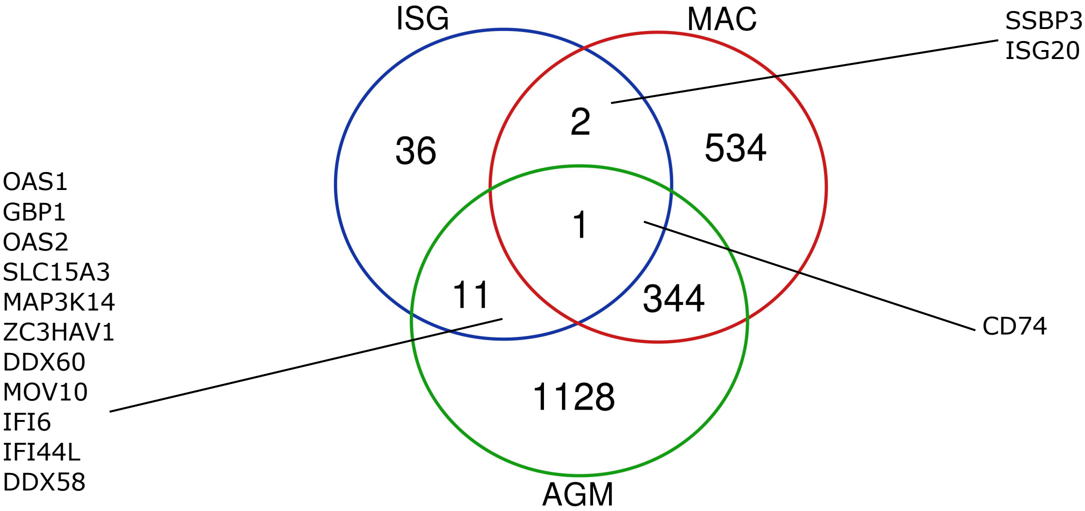

Comparison of the differentially expressed transcripts in CD32+ CD4+ T cells from the spleen of chronically SIV-infected MAC (red circles), AGM (green circles) with ISG genes (blue circle).

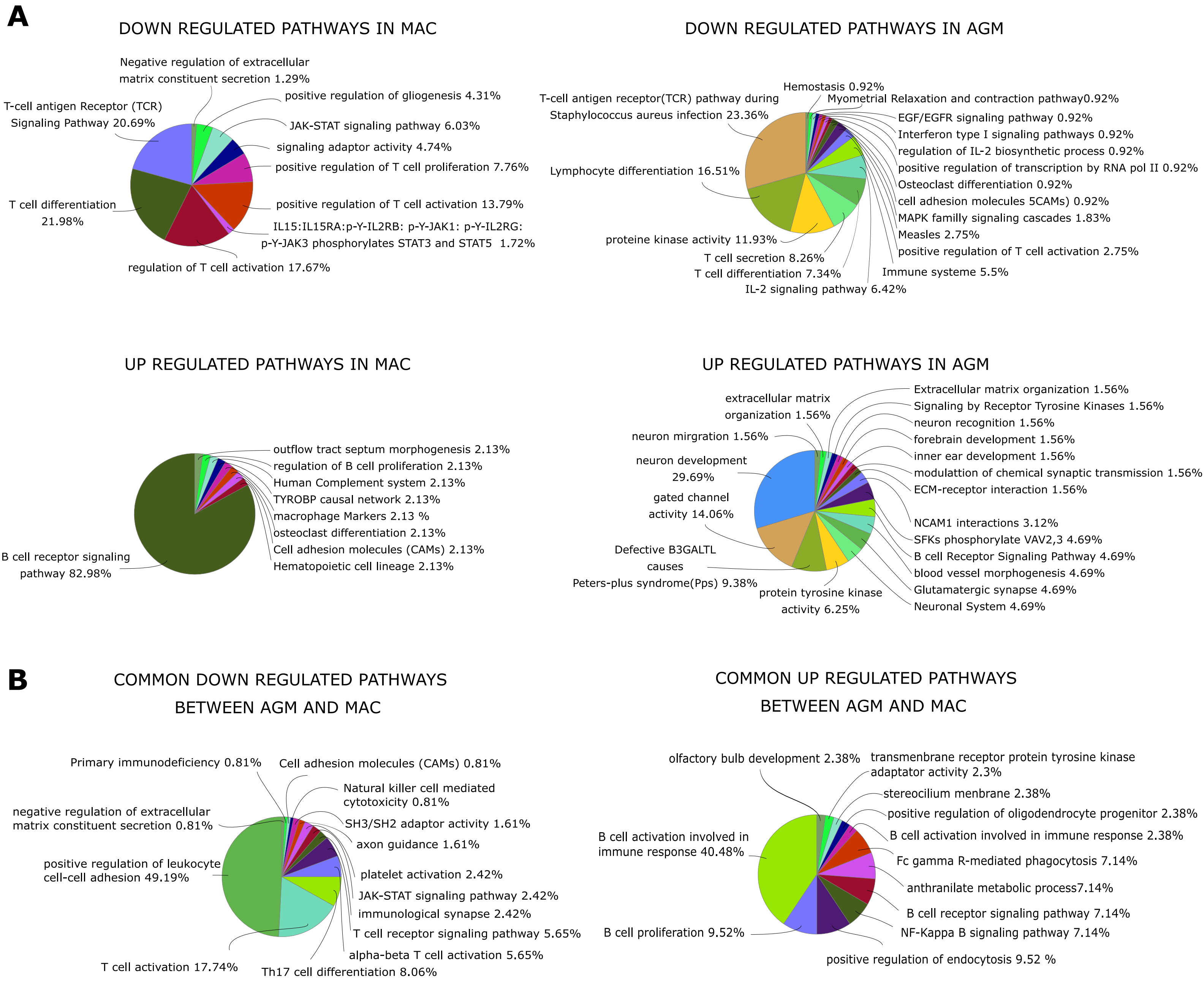
A
DOWN REGULATED PATHWAYS IN MAC
Negative regulation of extracellular matrix constituent secretion 1.29%
positive regulation of gliogenesis 4.31%
T-cell antigen Receptor (TCR) Signaling Pathway 20.69%
JAK-STAT signaling pathway 6.03%
signaling adaptor activity 4.74%
positive regulation of T cell proliferation 7.76%
T cell differentiation 21.98%
positive regulation of T cell activation 13.79%
IL15:IL15RA:p-Y-IL2RB: p-Y-JAK1: p-Y-IL2RG: p-Y-JAK3 phosphorylates STAT3 and STAT5 1.72%
regulation of T cell activation 17.67%
DOWN REGULATED PATHWAYS IN AGM
Hemostasis 0.92%
T-cell antigen receptor(TCR) pathway during Staphylococcus aureus infection 23.36%
Myometrial Relaxation and contraction pathway0.92%
EGF/EGFR signaling pathway 0.92%
Interferon type I signaling pathways 0.92%
regulation of IL-2 biosynthetic process 0.92%
Lymphocyte differentiation 16.51%
positive regulation of transcription by RNA pol II 0.92%
Osteoclast differentiation 0.92%
cell adhesion molecules 5CAMs) 0.92%
MAPK familly signaling cascades 1.83%
proteine kinase activity 11.93%
Measles 2.75%
positive regulation of T cell activation 2.75%
T cell secretion 8.26%
T cell differentiation 7.34%
Immune systeme 5.5%
IL-2 signaling pathway 6.42%
UP REGULATED PATHWAYS IN MAC
outflow tract septum morphogenesis 2.13%
regulation of B cell proliferation 2.13%
Human Complement system 2.13%
TYROBP causal network 2.13%
macrophage Markers 2.13 %
osteoclast differentiation 2.13%
B cell receptor signaling pathway 82.98%
Cell adhesion molecules (CAMs) 2.13%
Hematopoietic cell lineage 2.13%
UP REGULATED PATHWAYS IN AGM
Extracellular matrix organization 1.56%
extracellular matrix organization 1.56%
Signaling by Receptor Tyrosine Kinases 1.56%
neuron recognition 1.56%
neuron mirgration 1.56%
forebrain development 1.56%
neuron development 29.69%
inner ear development 1.56%
modulattion of chemical synaptic transmission 1.56%
ECM-receptor interaction 1.56%
gated channel activity 14.06%
NCAM1 interactions 3.12%
SFKs phosphorylate VAV2,3 4.69%
Defective B3GALTL causes Peters-plus syndrome(Pps) 9.38%
B cell Receptor Signaling Pathway 4.69%
blood vessel morphogenesis 4.69%
Glutamatergic synapse 4.69%
protein tyrosine kinase activity 6.25%
Neuronal System 4.69%
B
COMMON DOWN REGULATED PATHWAYS BETWEEN AGM AND MAC
Primary immunodeficiency 0.81%
Cell adhesion molecules (CAMs) 0.81%
Natural killer cell mediated cytotoxicity 0.81%
negative regulation of extracellular matrix constituent secretion 0.81%
SH3/SH2 adaptor activity 1.61%
axon guidance 1.61%
positive regulation of leukocyte cell-cell adhesion 49.19%
platelet activation 2.42%
JAK-STAT signaling pathway 2.42%
immunological synapse 2.42%
T cell receptor signaling pathway 5.65%
alpha-beta T cell activation 5.65%
T cell activation 17.74%
Th17 cell differentiation 8.06%
COMMON UP REGULATED PATHWAYS BETWEEN AGM AND MAC
olfactory bulb development 2.38%
transmenbrane receptor protein tyrosine kinase adaptator activity 2.3%
stereocilium menbrane 2.38%
positive regulation of oligodendrocyte progenitor 2.38%
B cell activation involved in immune response 2.38%
B cell activation involved in immune response 40.48%
Fc gamma R-mediated phagocytosis 7.14%
anthranilate metabolic process7.14%
B cell receptor signaling pathway 7.14%
B cell proliferation 9.52%
NF-Kappa B signaling pathway 7.14%
positive regulation of endocytosis 9.52 %

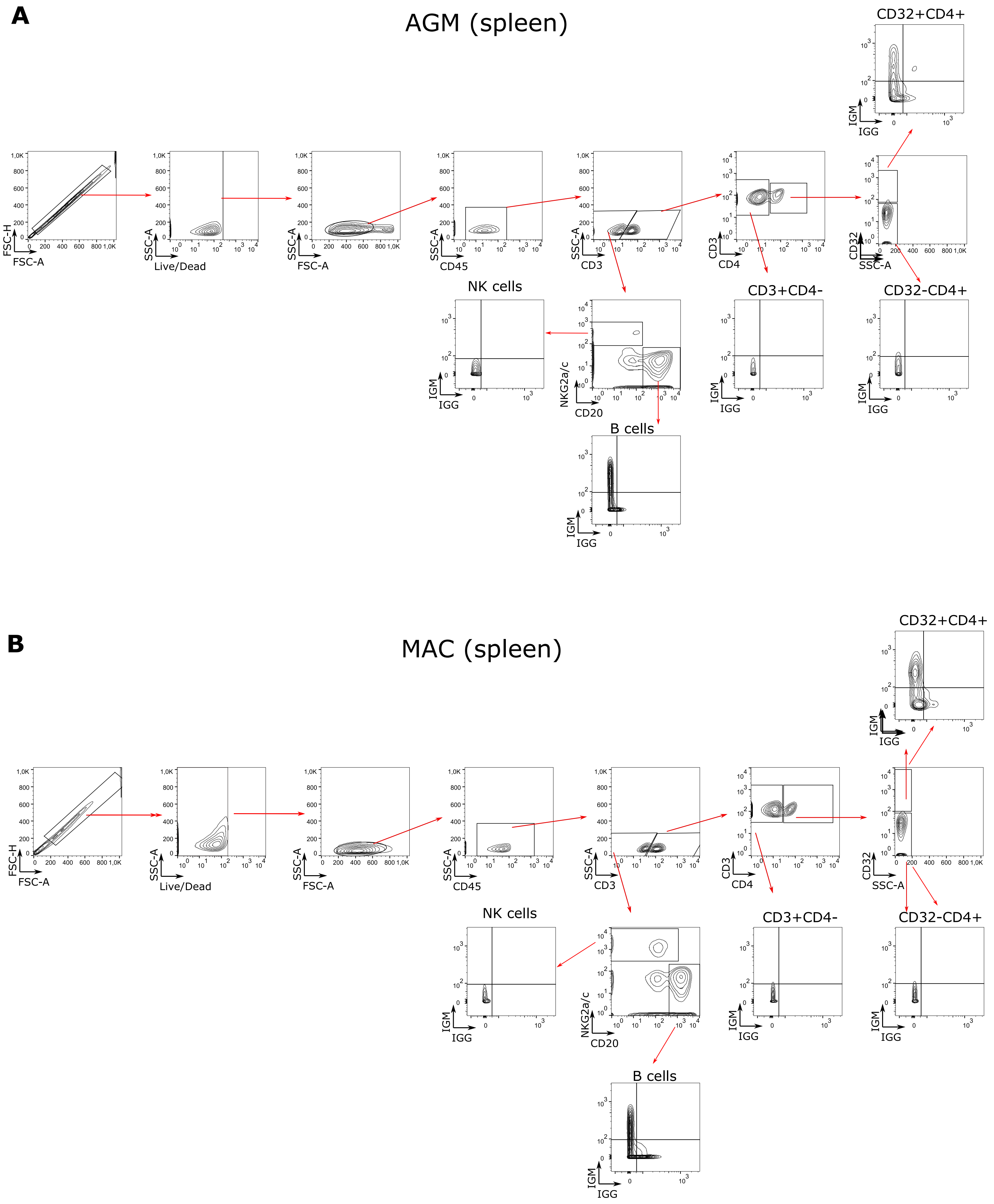
A
AGM (spleen)
FSC-H
FSC-A
SSC-A
Live/Dead
CD45
CD3
CD4
CD32
NK cells
NKG2a/c
CD20
B cells
CD3+CD4-
CD32+CD4+
CD32-CD4+
IGM
IGG
B
MAC (spleen)
FSC-H
FSC-A
SSC-A
Live/Dead
CD45
CD3
CD4
CD32
NK cells
NKG2a/c
CD20
B cells
CD3+CD4-
CD32+CD4+
CD32-CD4+
IGM
IGG
